# Screening and characterization of sex-specific sequences through 2b-RAD sequencing in American shad (*Alosa sapidissima*)

**DOI:** 10.1371/journal.pone.0282165

**Published:** 2023-03-02

**Authors:** Jia Du, Qinghua Liu, Yuhong Zheng

**Affiliations:** 1 Suzhou Fishseeds Bio-Technology, Suzhou, Jiangsu, China; 2 College of Materials and Environmental Engineering, Hangzhou Dianzi University, Hangzhou, Zhejiang, China; 3 Wisdom Lake Academy of Pharmacy, Xi’an Jiaotong-Liverpool University, Suzhou, Jiangsu, China; 4 Suzhou Health-Origin Bio-Technology, Suzhou, Jiangsu, China; Shanghai Ocean University, CHINA

## Abstract

American shad (*Alosa sapidissima*), introduced from the United States, has become one of the most expensive farmed fish in the aquatic product market of China. The shad reveals significant sexual dimorphism in growth and behaviors. For the study, five male-specific tags were identified in two-generation breeding populations of *Alosa sapidissima* and were verified by PCR amplification. Averages of 10,245,091 and 8,685,704 raw and enzyme reads were obtained by high-throughput sequencing of the 2b-RAD library, respectively. 301,022 unique tags were obtained from the sequences of twenty samples with sequencing depths of 0 to 500. Finally, 274,324 special tags and 29,327 SNPs were selected with a sequencing depth of 3 to 500. Eleven preliminary screening male-specific tags and three male heterogametic SNP loci were isolated. After verification by PCR amplification, five male-specific sequences of 27 bp located on chromosome 3 were screened out. Chromosome 3 could be assumed to be the sex chromosome of *Alosa sapidissima*. Sex-specific markers will provide invaluable and systematic animal germplasm resources to allow for the precise identification of neo-males for the all-female breeding of *Alosa sapidissima* in commercial aquaculture.

## Introduction

The growth indicators and time of sexual maturity are significantly different between sexes in fish species [1,2]. Culturing single-gender populations will improve both the commercial value and production of fish in aquaculture [2,3]. Since some farmed fish show sexual dimorphism in many characteristics, such as growth indicators or sexual maturity, understanding the sex determination system of these fish will help the aquaculture industry produce mono-sex fish. Genetic mechanisms of determining sex are diverse in fishes [4]. Male heterogamy (XX/XY) and female heterogamy (ZZ/ZW) are two kinds of sex determination systems in fish. The sex determination systems of XX/XO and ZO/ZZ have been found in fish these years [2]. Studying the sex determination systems and selecting sex-specific tags are beneficial for performing mono-sex control breeding [5]. Traditional technologies (amplified fragment length polymorphism (AFLP), simple sequence repeats (SSRs) [6] and random amplified polymorphic DNA fingerprinting (RAPD) [7] have been successful in identifying different sex-specific markers in some aquaculture fishes [8]. However, conventional methods are inefficient and time-consuming. Therefore, next-generation sequencing (NGS) technology has become widespread and provided efficient sex-related markers. Sex-specific DNA fragments and single nucleotide polymorphisms (SNPs) in vertebrates are usually identified by restriction site-associated DNA sequencing (RAD-seq), which is considered an efficient and time-saving technology. The sex-specific markers have played an essential role in the sex identification of fish, such as the zig-zag eel (*Mastacembelus armatus*), redtail catfish (*Mystus wyckioides*), Ussuri catfish (*Pseudobagrus ussuriensis*) and Nile tilapia (*Oreochromis niloticus*) [9–13].

American shad (*Alosa sapidissima*) is an essential fishery species naturally distributed in North America [14]. The shad were introduced to China in 2003 and successfully cultured in freshwater indoor systems for nearly 20 years in China [15]. The shad has become one of the most expensive farmed species in China, which has prompted an upsurge in consumption [16]. However, shad has quite distinctive dimorphism in body size and behaviors between sexes. American shad males have less commercial value due to both a smaller body size and growth rate than females [15]. Males are trouble makers that have vital chasing behaviors during spring seasons, causing severe body damage to the vulnerable fish and weight loss. Thus, it is crucial to develop an all-female culture population to take advantage of female growth superiority and avoid the risk of injury and weight loss caused by the sexual chasing behavior of males. Therefore, developing sex identification technology for identifying neo-males from sexually reversed mixed males has become a critical measure to develop an all-female population to increase economic output in shad aquaculture.

For the study, 2b-RAD-seq from 60 samples was carried out to show the sexual differences at the molecular level. Finally, five male-specific genotype tags were identified by PCR testing in many farmed mature fish. The research will supplement the data for all-female breeding and sex-specific gene in American shad.

## Materials and methods

### Sample collection

American shad was sampled from Suzhou Fishseeds Biotechnology in Suzhou, China. Ten males and ten females at 20 months old from the broodstock were randomly selected in August 2020 to identify by 2b-RAD sequencing. Twenty males and twenty females of the second generation were randomly sampled for marker validation in June 2021. All surgery was performed under sodium pentobarbital anesthesia, and all efforts were made to minimize suffering. The testis, ovaries, and muscle tissue were quickly taken by the surgery after anesthesia. The sex of individuals was determined by sexual organs after anatomy. Muscular tissues were sampled and stored at -20 °C. The total genomic DNA was extracted using Tissue DNA Extraction Kit (Catalog Number DR0301050). Then agarose electrophoresis and UV spectrophotometry (Hangzhou Youmi, Unano-1000) were used to test DNA quality.

### 2b-RAD library preparation and data analysis

Qingdao OE Biotech Co., Ltd. carried out the analysis of the 2b-RAD library (Qingdao, China), as referred to Wang et al. (2016) [17]. The details of library preparation and data analysis were shown in S1 Text.

### Screening of sex-specific sequences

The preliminary screening male-specific tags were 27 bp, which was not long enough for primer design. A chromosomal-level genome assembly of American shad was performed in our previous studies. The genome assembly number described in this paper is JAHTKL010000000 in NCBI. According to the location of the tag, the upstream and downstream 300–500 bp sequences of the tag were extracted and the location of the tag was marked. Finally, the primers were designed based on the upstream and downstream 300–500 bp of the tag.

### Identification of sex-specific markers

This experiment referred to Zhu et al., 2021 [12]. 14 pairs of primers (Premier 5.0 software) were designed according to eleven sex-specific markers and three sex-specific SNPs (shown in S1 Table) to identify the male-specific 2b-RAD markers. Firstly, PCR amplified was based on fourteen pairs of primers in twenty fish in F0 generation. Then the validated primers were further amplified in forty fish in the second generation. The details of the total PCR volume and amplification program were shown in S2 Text.

### Example ethics statement

This study was carried out in strict accordance with the recommendations in the Guide for the Care and Use of Laboratory Animals of the National Institutes of Health. The protocol was approved by the Laboratory Animal Ethics Committee of Suzhou Jianyuan Technology Co., LTD. (Protocol Number: 14393). All surgery was performed under sodium pentobarbital anesthesia, and all efforts were made to minimize suffering.

## Results

### The data of 2b-RAD sequencing

**Fig 1:**
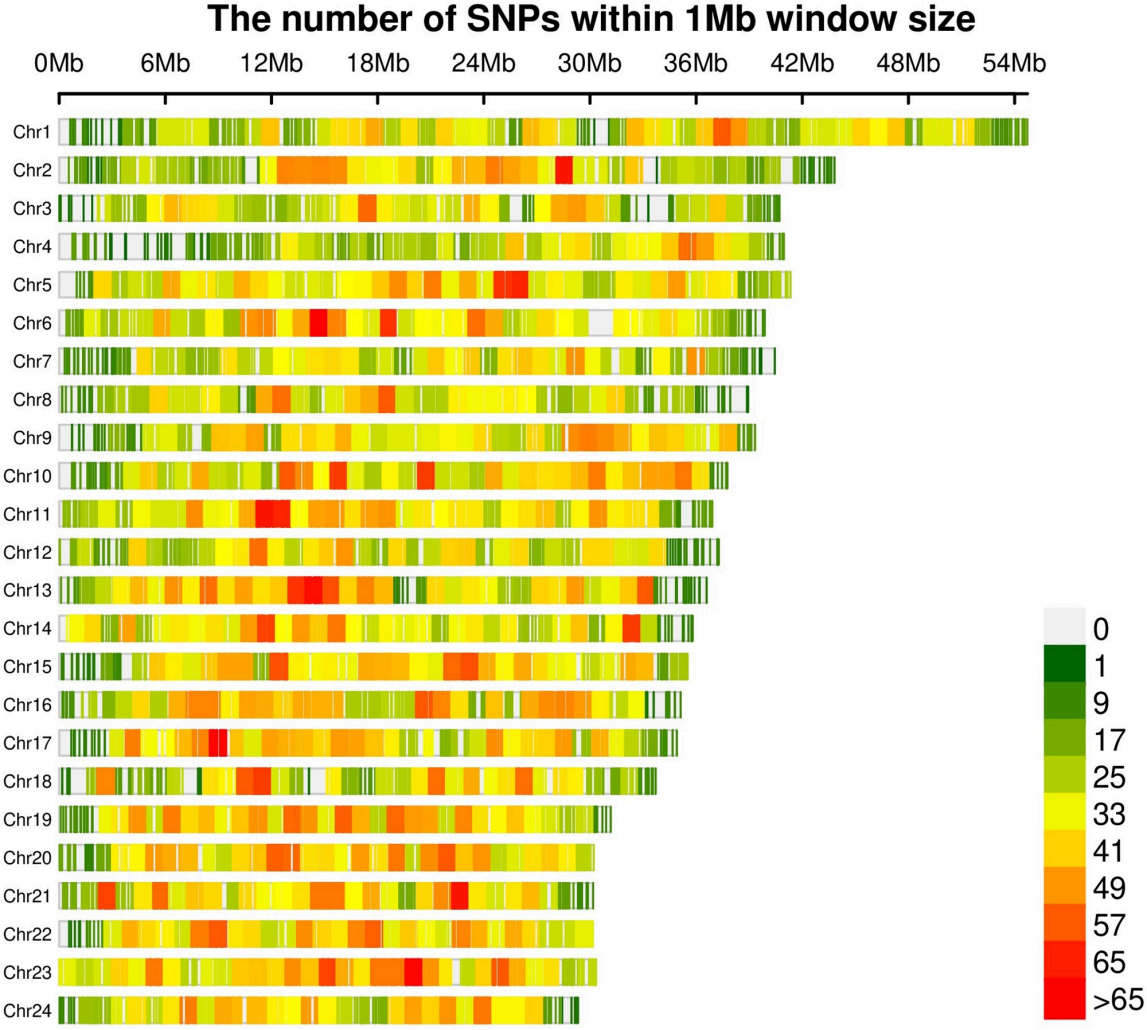
Distribution diagram of SNP markers in the chromosomal map.

The genome sequences of *Alosa sapidissima* (version JAHTKL010000000) were chosen as the reference genome. After high-throughput sequencing, averages of 10,245,091 and 8,685,704 raw and enzyme reads were obtained, respectively (shown in Table 1). 301,022 unique tags with sequencing depths of 0 to 500 were obtained. 274,324 tags and 29,327 SNPs were obtained after the removal of unique tags with a sequencing depth of 3 to 500. The distribution of the SNPs on the chromosomes is shown in Fig 1. The proportion of unique tags ranged from 65.07% to 67.41% per sample, and the average sequencing depth was 20.77× (3<depth<500). The result reveals valuable genomics data for identifying sex-specific tags and SNPs.

**Table 1:** Summary of 2b-RAD data obtained from 20 *Alosa sapidissima* individuals.

Name	Sex	Raw Reads	Enzyme Reads	Percentage	Tags(depth>0)	Sequencing depth(depth>0)
F-1	Female	9,635,733	8,503,555	88.25%	300558	18.76
F-2	Female	9,635,733	8,594,215	89.19%	301003	18.8
F-3	Female	9,635,733	8,508,372	88.30%	295568	18.83
F-4	Female	9,635,733	8,565,042	88.89%	301770	18.59
F-5	Female	9,635,733	8,135,190	84.43%	302209	18.15
F-6	Female	9,709,760	8,260,097	85.07%	303939	18.24
F-7	Female	9,709,760	8,540,728	87.96%	301313	18.98
F-8	Female	9,709,760	8,520,089	87.75%	296344	19.17
F-9	Female	9,709,760	8,219,843	84.66%	291952	18.61
F-10	Female	9,709,760	8,246,326	84.93%	300919	17.83
M-1	Male	11,392,023	9,504,713	83.43%	303886	20.82
M-2	Male	11,392,023	9,299,567	81.63%	300126	20.45
M-3	Male	11,392,023	9,161,422	80.42%	297416	20.56
M-4	Male	11,392,023	9,002,391	79.02%	298414	19.81
M-5	Male	11,392,023	9,080,605	79.71%	305348	19.91
M-6	Male	10,242,846	8,746,277	85.39%	307324	19.05
M-7	Male	10,242,846	8,917,236	87.06%	304808	19.17
M-8	Male	10,242,846	8,621,828	84.17%	298935	19.34
M-9	Male	10,242,846	8,637,325	84.33%	303830	19.01
M-10	Male	10,242,846	8,649,257	84.44%	304774	18.49
Average		10,245,091	8,685,704	84.95%	301022	19.12

### Identification of sex-specific markers and SNPs

**Fig 2:**
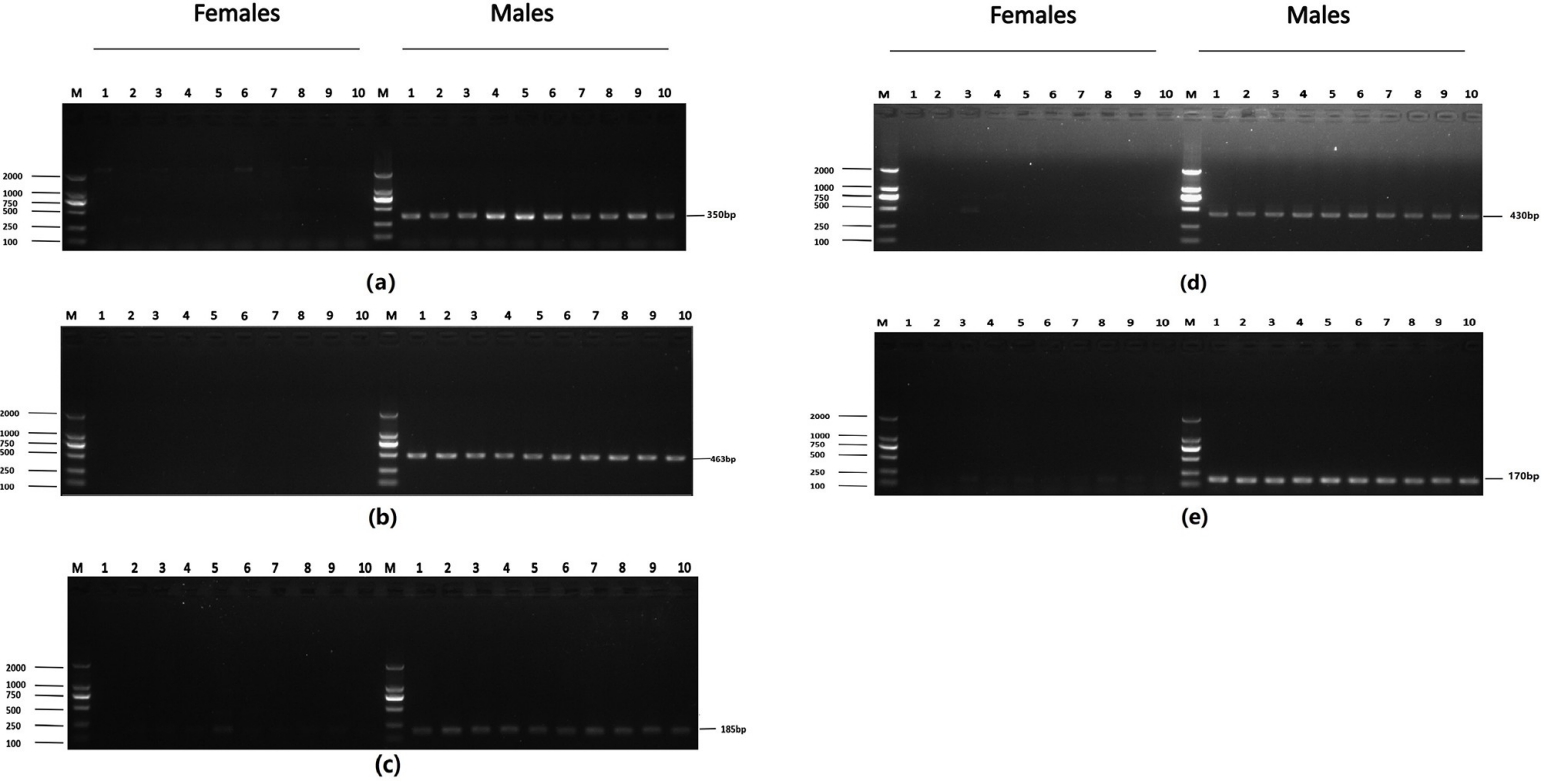
Identification of five male-specific tags: (a) DNA fragment amplified with Tag8736997; (b) DNA fragment amplified with Tag8743751; (c) DNA fragment amplified with Tag8821742; (d) DNA fragment amplified with Tag8818409; (e) DNA fragment amplified with Tag8821444; the left shows DL 2000 DNA marker sizes; the right shows fragment sizes.

The preliminary screening of eleven male-specific tags (Table 2) and three heterogametic SNP loci (Table 3) were obtained. The male specificity of the preliminary screening sex-specific markers was verified by a basic local alignment search tool (BLAST) search of the *Alosa sapidissima* genome. The result showed that the preliminary screening tags and SNP loci revealed 100% match to the reference gene. The male-specific sequences were identified and compared with the assembled genome sequence of male *Alosa sapidissima* (JAHTKL010000000) obtained from NCBI by BLAST. Then, 14 pairs of primers were designed to test the preliminary screening markers and SNPs (S1 Table). Finally, five male-specific tags were confirmed by PCR in twenty samples. The five male-specific sequences with lengths of 350 bp, 463 bp, 430 bp, 170 bp and 185 bp are shown in Table 4. The male-specific primers of five tags (Tag8736997, Tag8743751, Tag8818409, Tag8821444 and Tag8821742) were shown in Table 5. The results indicated that the male-specific primers of five tags could only amplify bands in male fish (Fig 2).

**Table 2:** Information of 11 male-specific 2b-RAD tags obtained from *Alosa sapidissima*.

Name	Ref ID	Sequences	Existence in the male genome not in the female genome
Tag-8728405	Chr3-8728405	AGAGGTTGGAGTTGGAGTTGGGAGGAT	Yes
Tag-8736997	Chr3-8736997	GAATAAAGGAGCAACAGTCTTCAGCAT	Yes
Tag-8743751	Chr3-8743751	ATAGCAACCACCAAATCTCCCCTAGCT	Yes
Tag-8801108	Chr3-8801108	AGTTGATGGAGGTGGTGTTGGGGGGTT	Yes
Tag-8812164	Chr3-8812164	TAAAGTGGGAGGGGCTGTAATGTACAA	Yes
Tag-8814109	Chr3-8814109	CAGCACTGGAGATGGGGTACTGTTTAA	Yes
Tag-8815382	Chr3-8815382	CATCGTGGGAGCCACTGTACTGAGGTG	Yes
Tag-8816193	Chr3-8816193	ACAATATGGAGTTGTCGTAAATCTAGA	Yes
Tag-8818409	Chr3-8818409	GCCATAATCACCCCTCCTCCTCTCCCT	Yes
Tag-8821444	Chr3-8821444	GACAGATGGAGGGGATGTGTGGGTGTA	Yes
Tag-8821742	Chr3-8821742	TTTCACCGGAGTTAAAGTATAGTGACA	Yes

**Table 3:** Information of 3 male-specific SNP obtained from *Alosa sapidissima*.

Name	Ref ID	Alleles of reference sequences	Alleles of variant sequences
SNP-9284363	Chr3-9284363	C	T
SNP-8878197	Chr3-8878197	T	C
SNP-8786815	Chr3-8786815	T	C

**Table 4:** Information of the nucleic acid sequence obtained from male Alosa sapidissima (JAHTKL010000000).

Male-specific 2b-RAD-tags	Sequences (5′−3′)	Size
Tag8736997	TCAGGCACATTCAGATCAGATCTCATCGGGGTAGCAGCTGCCACTCATCACTCTATTGCCTGACGGTCCAGTCCTGACGCTGTTTGGATTGTATTCTCACTGCAATACGGTGGAATAAAGGAGCAACAGTCTTCAGCATTCTCGTCTTGGTGTTCTCCTTCGGGTCTTTGACATCAAAGTGCTAGTTGGATTGGAGGTTCGGGAAAGTGGATAGTTTTTCCACGACAAAGCTCCCAGTTAGCTATATTGCCAGTTTTGTGGGCCACTTACAGACACAAAATGCAATTGAAATTTCGGTGCAACATGAACAAGAAGATGCATTTAACGTTGAGCCCCTAGGGATCAAGTAT	350bp
Tag8743751	AAATGATTACCCTAGCAACCAGCATAGCAACCACTTTATTTAGCAATAGCAACCACCAAATCTCCCCTAGCTAGTAGCCTAAATCATATATTTTTGGAATGCACTCAGATATATTGTACATTCAAAGATCAACTGGACAGCTGTATCTTACCACCCAGATAGCAGACTTTCTGGCAGCACTTTGGCATACATCTGGCGATTTTGGGCTTTGATATGGCAGACCAAAACTTTTGGCTTCAATGTGGCATGATTATGGGCAGCCATAACAGATAAGAAGGCGCCAGTAGTGTATGGCTGAATTATGGCATATATCTGGTCAACCGTAGCGTTAGATCAAGAGCGGGGACAGCATCTGATGACGCATACAGGCTGTTTGGCATGTTTATGGACAGCCAGAAGATGGGCGAAGAGCGGGACAGAGTCTACCCGTATGTGGGTGAGTTCTGGCATGTTTCTGGCTAAC	463bp
Tag8818409	TTTCCTTCTCCCTGCTTACAAATGCAAACCCCATTTTGTTTTCTCCGAAGTGAAGATGCTGATGATGAAGACTGCTCGGAGGGCCCTGACAGGGATGAAGACTGACAGGACACCCAGGGAGGCAGCCTGGCATTGACCGGCCTATCCTTTAATGCCGTGGCCATAATCACCCCTCCTCCTCTCCCTGCTTCCCTCTGAGTGGTGGGCAGTGCCAACATGTCACTTAAAGGCCAGTTGGCAGCGTCCCCGACAGTGGGGCCTGCATGTGTCACGTGCGCTGATTTGGCTAGCCACCCAGCCTAGTGGATGAGAGAGGGAGGGGTCAGTCTTGAAGGCTGTTCACACCAGGAATGATAACTATAAAAATAATGGCAAAAACAACAGTCCGCTCCAGCTAATATGAAGGACAACGTCCACACCACATACTACG	430bp
Tag8821444	GGCGTAATTTCAATGGAAAGTGACTTCCAAACCCCCCAAAACCCACTAAATCGTGGACCCTAGTCTTCAAACAGCGTGAGGCCATTGGACAGATGGAGGGGATGTGTGGGTGTACGTAGTGCAGTTTCCAGAATTAGACCTCTGGTCTCCACCAGGAGAGAGATCATCTC	170bp
Tag8821742	AAGGGTTAACAAGGGTGAGAGAGGAGAAATATGTGTGTGTGTGTGTGTGAGAGTTTTCACCGGAGTTAAAGTATAGTGACAAAGAGATCTAGAAATAGAGGGAGAAATGAATGAAAAGAAGAGAAGAGAGACAGAGAGAGAAAGAGAGAGAGAGACAGTCTGTTAACTTCACGTTTGGCCTCAGT	185bp

**Table 5:** Primers of male-specific 2b-RAD-tags.

Male-specific 2b-RAD-tags	Primer sequences (5′−3′)	Product Size (bp)	Tm (◦C)
Tag8736997	F-TCAGGCACATTCAGATCAGATCTC R-ATACTTGATCCCTAGGGGCTCAAC	350	62.891
Tag8743751	F-AAATGATTACCCTAGCAACCAGCA R-GTTAGCCAGAAACATGCCAGAACT	463	63.043
Tag8818409	F-TTTCCTTCTCCCTGCTTACAAATG R-CGTAGTATGTGGTGTGGACGTTGT	430	62.811
Tag8821444	F-GGCGTAATTTCAATGGAAAGTGAC R-GAGATGATCTCTCTCCTGGTGGAG	170	62.937
Tag8821742	F-AAGGGTTAACAAGGGTGAGAGAGG R-ACTGAGGCCAAACGTGAAGTTAAC	185	63.076

### Verification of male-specific tags in F1 generation

**Fig 3:**
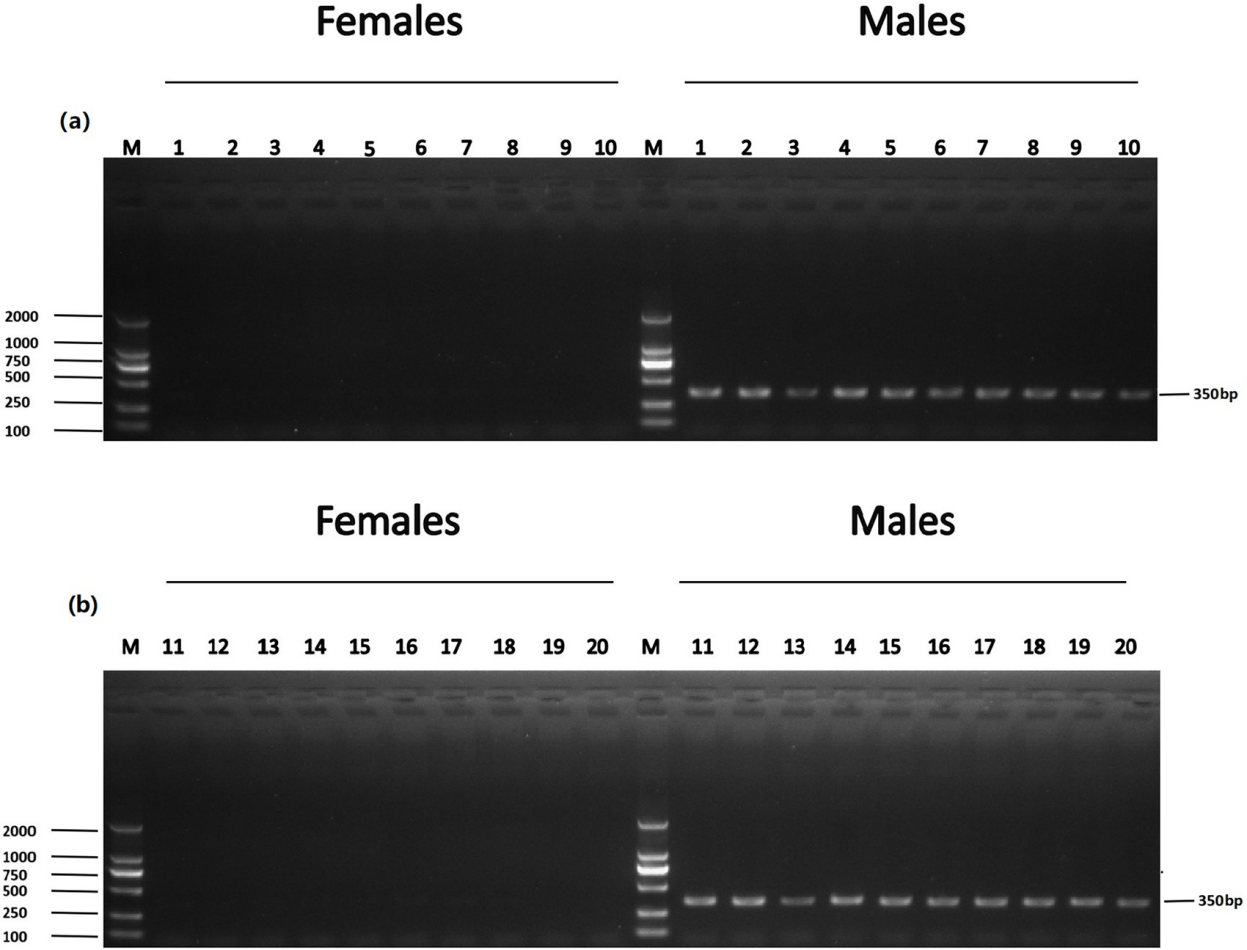
Identification of a male-specific tag, DNA fragment, amplified with Tag8736997. The left shows DL 2000 DNA marker sizes; the right shows fragment sizes.

**Fig 4:**
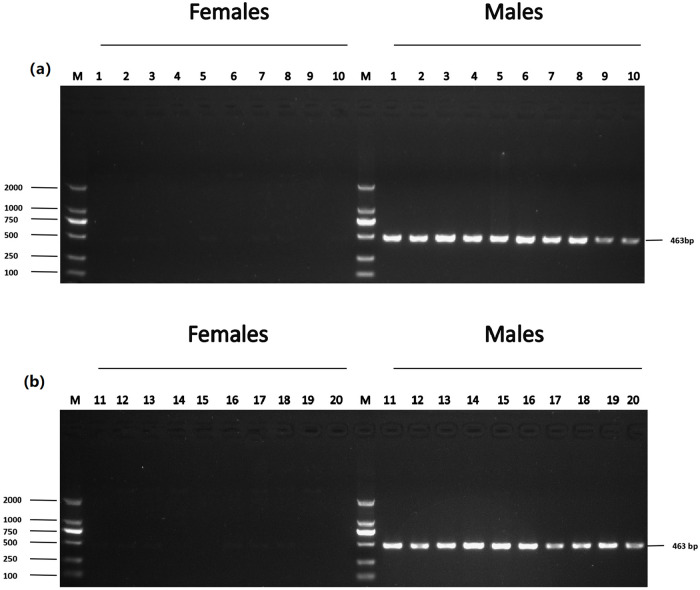
Identification of a male-specific tag, DNA fragment, amplified with Tag8743751. The left shows DL 2000 DNA marker sizes; the right shows fragment sizes.

**Fig 5:**
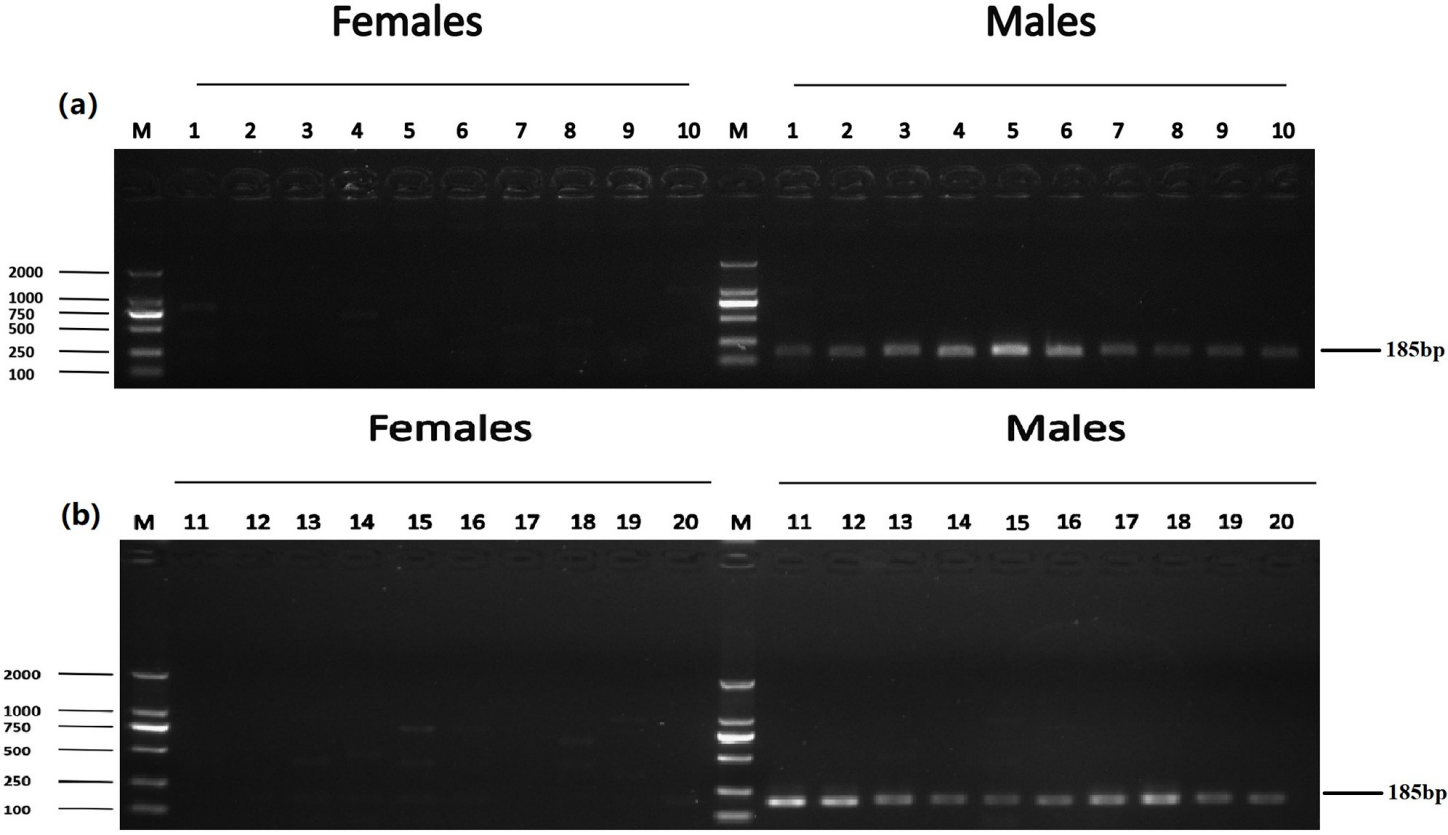
Identification of a male-specific tag, DNA fragment, amplified with Tag8821742. The left shows DL 2000 DNA marker sizes; the right shows fragment sizes.

**Fig 6:**
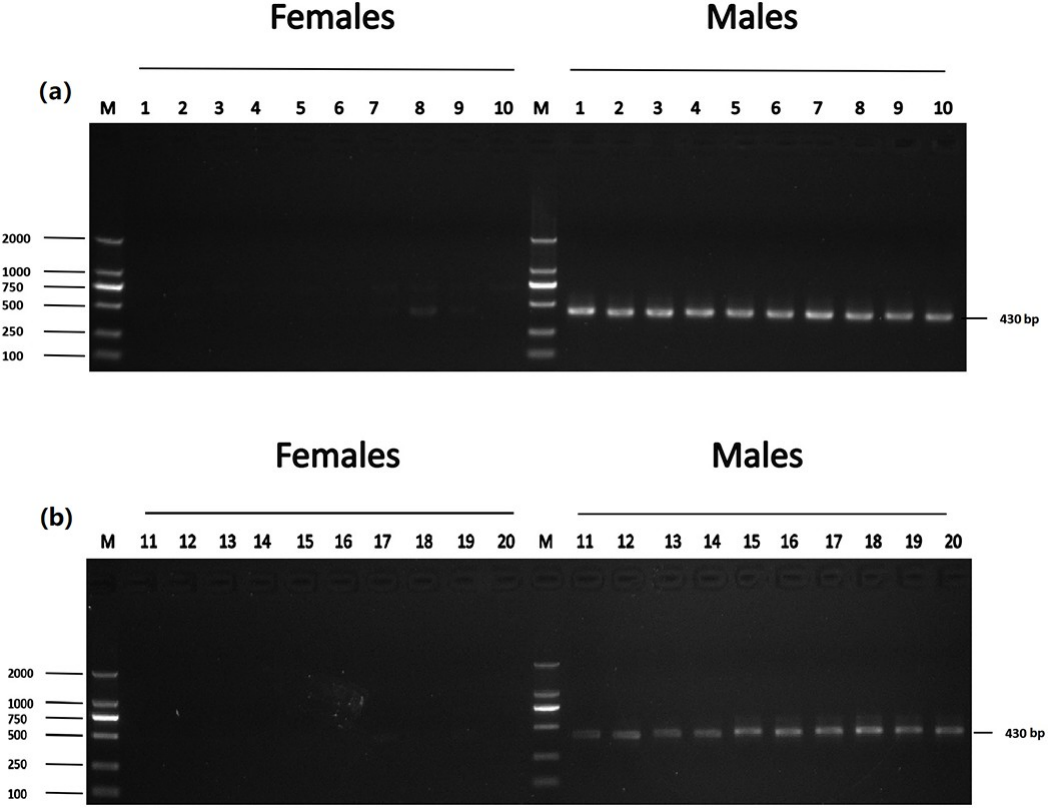
Identification of a male-specific tag, DNA fragment, amplified with Tag8818409. The left shows DL 2000 DNA marker sizes; the right shows fragment sizes.

**Fig 7:**
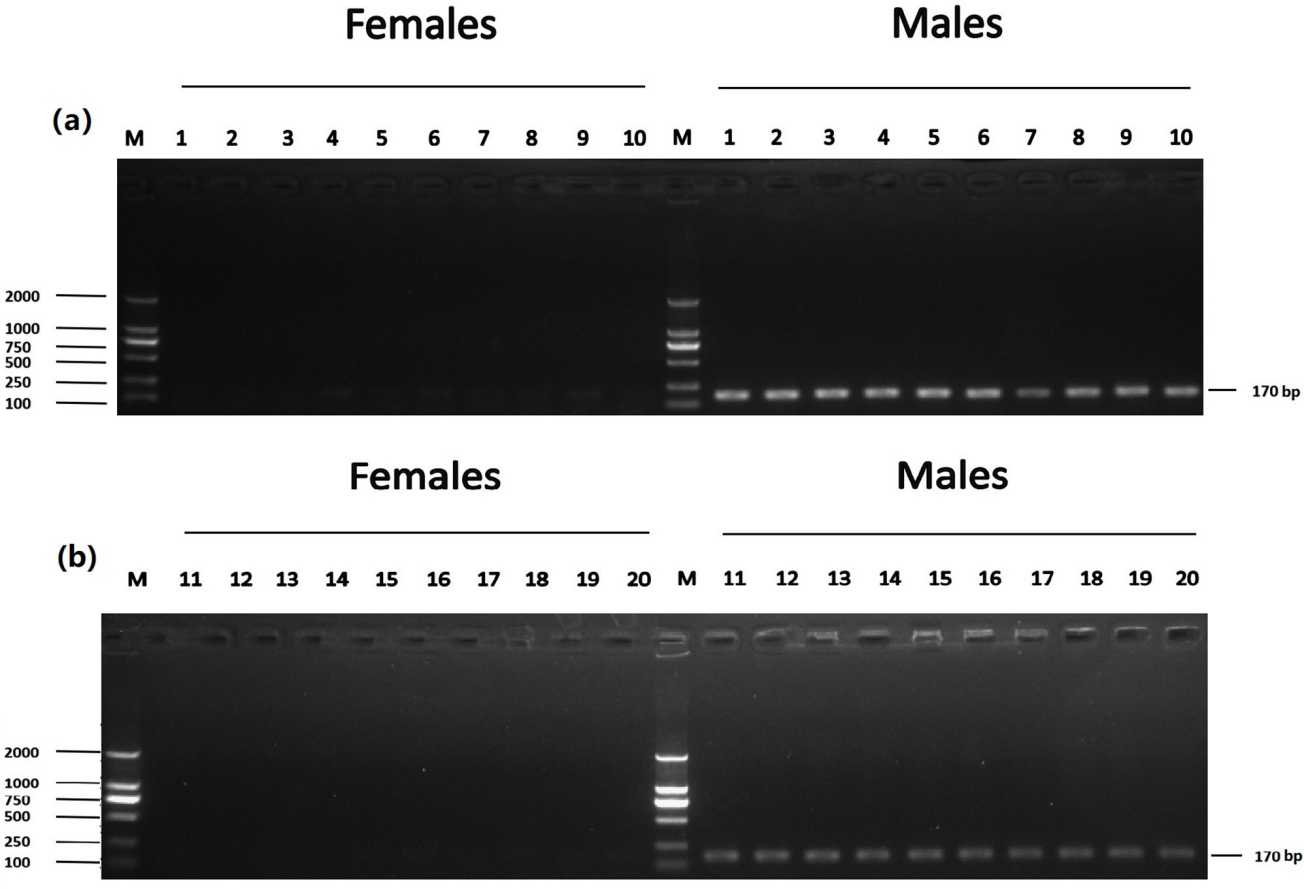
Identification of a male-specific tag, DNA fragment, amplified with Tag8821444. The left shows DL 2000 DNA marker sizes; the right shows fragment sizes.

The accuracy of five male-specific tags was identified by the F1 generation. Twenty male and twenty female samples with sex confirmed by sexual organs were obtained for PCR validation. As a result, the male-specific 350 bp, 463 bp, 185 bp, 430 bp and 170 bp sequences were amplified in male fish by the primers Tag8736997 (Fig 3), Tag8743751 (Fig 4), Tag8821742 (Fig 5), Tag8818409 (Fig 6) and Tag8821444(Fig 7), respectively. But these verified male-specific tags need further identification in wild *Alosa sapidissima*. However, this research can supplement genetic information for mono-sex control breeding in *Alosa sapidissima*.

## Discussion

For aquaculture, sex-controlled breeding and monosex culturing are essential to ensure both yield and nutrient requirements. Sex determination systems in fishes are various, with heterogametic males (XY) or heterogametic females (ZW) [18]. Determining the sex determination system in fishes is difficult because of the highly similar size and low differentiation of heteromorphic sex chromosomes [19]. In recent years, unisexual cultivation has developed rapidly due to the development of sex-specific markers [20]. Sex determination genes and differentiation pathways can help researchers screen the sex-specific markers in some aquaculture fishes [21]. For instance, sex-specific markers were found from *dmrt1* gene of the yellow drum (*Nibea albiflora*), amhr2 gene of Patagonian pejerrey (*Odontesthes hatcheri*) and *amhr2* gene of pufferfish (*Takifugu obscurus*) [22–24]. Conventional technologies, such as AFLP and RAPD, have already screened sex-specific markers in some aquatic species [25,26]. However, conventional technologies are time-consuming, and cannot detect all the sex-specific markers in fishes [26]. Consequently, high-throughput methods of NGS technology are the best choice to verify sex-specific markers and sequences.

The sex-specific markers of American shad are still unclear. In the research, we created an efficient testing process to identify male-specific tags and male-specific SNPs. Male-specific tags were identified in 60 samples of two generations of fish. Sex-specific markers can help researchers identify the sex-related genes in American shad. American shad is an essential economic fish species and widely farmed in China. However, the culture model of all female fish increases the production efficiency of American shad. The verification of sex-specific markers can help promote the process of genetic breeding [27]. The technology of mono-sex breeding for the mass production of economic fish is developing rapidly [28]. In this context, sex-specific molecular markers of American shad are urgently needed. All-female production technology has been developed and successfully applied in yellow catfish [29–31]. However, sex determination is a complex process, which has its unique evolutionary patterns and it also can be affected by external factors, including genetic, environmental (e.g., temperature), behavioral, and physiological factors [32]. For example, Baroiller et al. (2009) found that the sex determination system of the Nile tilapia (*Oreochromis niloticus*), both domestic and wild populations, was affected by the interactions between genetic determination and temperature [33]. We can achieve sex reversal through hormone feeding and breed XX neo-males, producing an all-XX female brood. In conclusion, five male-specific tags are identified, and the XX/XY sex system of American shad is predicted. The results of PCR and agarose gel electrophoresis were validated in two generations. However, the sex-specific markers of American shad are still unclear. The sex determination and differentiation pathway genes associated with the five male-specific tags should be identified. Those genes combined with sex-specific markers are important for developing sex-controlled breeding to generate all-female populations in the aquaculture industry.

## Conclusions

This is the first study to identify sex-specific markers using 2b-RAD sequencing in American shad. The preliminary screening of eleven male-specific tags (Table 2) and three male heterogametic SNP loci were screened in twenty fish using 2b-RAD sequencing. The male-specific sequences were used to design sex-specific primer pairs, and five tags were finally identified by PCR in the second generation. The results indicate that five male-specific tags can accurately identify sex in American shad. The research created an efficient technological means for identifying the genetic sex of American shad and for the quantity production of all-female fish.

## Supporting information

S1 Table Primers used to verify candidate male-specific 2b-RAD-tags and sex-specific SNP locus. (DOCX)

S1 Text The details of library preparation and data analysis. (DOCX)

S2 Text The details of the total PCR volume and amplification program. (DOCX)

S1 Raw images (DOCX)
