# Supplementary material for: Screening and characterization of sex-specific sequences through 2b-RAD sequencing in American shad (Alosa sapidissima)
Source: PLoS One. 2023 Mar 2;18(3):e0282165. doi: 10.1371/journal.pone.0282165 (PMC9980781; doi:10.1371/journal.pone.0282165)
Supplement: S1 Table — (DOCX) [file pone.0282165.s001.docx]

**S3 Table Primers used to verify candidate male-specific 2b-RAD-tags and sex-specific SNP locus**

| Male‐specific  2b‐RAD‐tags | Primer sequences (5′−3′) | Product Size (bp) | Tm (◦C) |
| --- | --- | --- | --- |
| Tag-8728405 | F-GCAGAGTGGTAAGCGTCTGGTAAT  R-CCAGTATCCTGTTCCTGACGAGAT | 467 | 62.898 |
| Tag-8736997 | F-TCAGGCACATTCAGATCAGATCTC  R-ATACTTGATCCCTAGGGGCTCAAC | 350 | 62.891 |
| Tag-8743751 | F-AAATGATTACCCTAGCAACCAGCA  R-GTTAGCCAGAAACATGCCAGAACT | 463 | 62.949 |
| Tag-8801108 | F-AACCGAGCTAAACACGTTTGCTAG  R-GTCAATCAATGAGCAACTCCCTCT | 490 | 63.017 |
| Tag-8812164 | F-GTTCATTAGTTCCCCTGTGCTGAC  R-TCATTATTGGGTTGATAGCAGGCT | 470 | 63.034 |
| Tag-8814109 | F-GGGGAGAACATCACTAAGGAACCT  R-ATCACAGGTACGGCCTTATGTGTT | 196 | 62.949 |
| Tag-8815382 | F-CATCGCTGCTGAAAGTAACTTCAA  R-TGTGGTATGAATAGCTGCAATGCT | 233 | 62.988 |
| Tag-8816193 | F-CTCTCCTTTCTGTGCATCATCCTT  R-CAACTTAAAGCCTTCCAGACAGGA | 479 | 63.015 |
| Tag8818409 | F-TTTCCTTCTCCCTGCTTACAAATG  R-CGTAGTATGTGGTGTGGACGTTGT | 430 | 62.811 |
| Tag8821444 | F-GGCGTAATTTCAATGGAAAGTGAC  R-GAGATGATCTCTCTCCTGGTGGAG | 170 | 62.937 |
| Tag-8821742 | F-AAGGGTTAACAAGGGTGAGAGAGG  R-ACTGAGGCCAAACGTGAAGTTAAC | 185 | 62.848 |
| SNP-9284363 | F-ACACACGGATACACACACACACAC  R-AGGATTCACATCTAGCCAGGAGTG | 386 | 63.014 |
| SNP-8878197 | F-TGGACTCGTCTTGTAATCCCTCTC  R-CACACCTGTCATCTGGAAATTGAC | 151 | 62.922 |
| SNP-8786815 | F-TCAATCTTCTGTTCGGCTAGTTCC  R-CTGTACTACGGCCCTGCAGTAAGT | 323 | 62.969 |
