# Supplementary material for: Screening and characterization of sex-specific sequences through 2b-RAD sequencing in American shad (Alosa sapidissima)
Source: PLoS One. 2023 Mar 2;18(3):e0282165. doi: 10.1371/journal.pone.0282165 (PMC9980781; doi:10.1371/journal.pone.0282165)
Supplement: S1 Text — (DOCX) [file pone.0282165.s002.docx]

For each sample, 100-200 ng genomic DNA was digested by 1 U BsaXI (New England Biolabs, cat. no. R0609) in a 15 µl reaction volume at 37 °C for 45 min. Four microliters of digested DNA (~50 ng) was run on a 1% agarose gel to verify the effectiveness of digestion. A total of 20 µl (Table 1) of ligation master mix containing 0.2 µM of each specific adaptor (five pairs of adaptors per five samples shown in (Table 2 and Table 3), 0.5 mM ATP (New England Biolabs), 200 U T4 DNA ligase (New England Biolabs), 1 µl 10 × T4 ligase buffer, 5.9 µl nuclease-free water, and 10 µl digestion product was used. Each reaction tube was incubated at 16 °C for one hour. The ligation products were amplified in a total volume of 50 µl in the PCR system (Table 4), each composed of each primer at 0.16 µM (Table 3 and 5), 0.24 mM dNTP, 10 µl 5× HF buffer, 0.8 U Phusion high-fidelity DNA polymerase (New England BioLabs), 18.8 µl nuclease-free water, and 18 µl ligation products. Amplification was performed using the following PCR program: PCR was conducted in MyCycler thermal cyclers (Bio-Rad) with 16 cycles of 98 °C for 5 s, 60 °C for 20 s, and 72 °C for 10 s. Fifty microliters of PCR product was run alongside 1 µl of 100-bp DNA ladder on an 8% (wt/vol) polyacrylamide gel at 400 V for 35 min. The gel was stained with SYBR Safe DNA stain for 3 min and then viewed briefly (<30 s) on a UV transilluminator set to verify the presence of the target bands (100 bp). The target band was cut out in a narrow gel slice and transferred to a 1.5-ml microcentrifuge tube and smashed using a pestle. Then, 30–40 µl of pure water was added and incubated at 37 °C for 30 min. The tubes were centrifuged at 14,000 × g for 2 min at room temperature. For each tube, 12 µl of the supernatant was used as a template, and the above PCR steps were repeated for 4-6 PCR cycles to improve the yield. PCR products from five samples were mixed, and the mixture was purified using a MinElute PCR Purification Kit. Thirty microliters of digestion master mix containing 1 mM ATP, 3 µl 10× CutSmart buffer, 2 U SapI (New England Biolabs), 10 µl purified mixed PCR product, and 13.8 µl nuclease-free water was prepared, and the mixture was incubated at 37℃ for 30 min. The digested product was added to a tube containing pretreated magnetic beads, and the mixture was incubated at room temperature. A magnet was applied, and the supernatant was transferred to a new tube. Then, 200 U T4 DNA ligase was added to the supernatant, and the mixture was incubated at 16 °C for 45 min. Gel purification was performed by the following steps: the PCR product was run alongside 1 µl of 100-bp DNA ladder on an 8% (wt/vol) polyacrylamide gel at 400 V for 35 min. The gel was stained with SYBR Safe DNA stain for 3 min and then viewed briefly (<30 s) on a UV transilluminator set to verify the presence of the target bands (100 bp). The target band was cut out in a narrow gel slice and transferred to a 1.5 ml tube. The size of the target band was 244 bp. The tube was centrifuged at 14,000 × g for 2 min at room temperature the supernatant was transferred to a new tube. Barcodes were introduced by PCR with barcode-bearing primers. PCR products were purified using a MinElute PCR Purification Kit and pooled for sequencing using the Illumina PE sequencing platform.

Then, 2b-RAD genotyping was performed with the RAD typing program v1.5 [1]. Adaptor sequences were removed from raw reads. The terminal 3-bp positions were excluded from each reading. After reads filtering, the SOAP software (version 2.21) [2] was used to aligned against high-quality reads and the known BsaXI sites in the American shad genome. Genotypes were assigned to sites using the maximum likelihood (ML) approach [1]. SNPs with minor allele frequencies of 5% were discarded. Based on the high-quality SNPs obtained, we developed sex-differentiated labels and SNP markers and finally obtained a short sequence specific to male shad that was present in all ten male shad but not in any of the ten female shad.

**Reference**

1. Fu, X., Dou, J., Mao, J., et al. RADtyping: An Integrated Package for Accurate De Novo Codominant and Dominant RAD Genotyping in Mapping Populations. PLoS ONE 8(11): e79960 (2013).
2. Li, R.Q., Li, Y.R., Kristiansen, K., Wang, J. SOAP: short oligonucleotide alignment program. Bioinformatics. 24, 713–714 (2008).

**Table 1** **Volume for PCR**

| Component | Volume (for single tag; µl) |
| --- | --- |
| Digestion product | 10 |
| 10× T4 ligase buffer | 1 |
| 10 m M ATP | 1 |
| Adaptor A (5µM) | 0.8 |
| Adaptor B (5µM) | 0.8 |
| T4 DNA ligase (400 U/µl) | 0.5 |
| Pure water | 5.9 |
| Total | 20 |

**Table 2 The adaptor combinations for the five tag positions**

| Tag position | Adaptor A | Adaptor B |
| --- | --- | --- |
| 1 | Ada 1 | Ada 2 |
| 2 | Ada 3 | Ada 4 |
| 3 | Ada 5 | Ada 6 |
| 4 | Ada 7 | Ada 8 |
| 5 | Ada 9 | Ada 10 |

| **Table 3 \|** The oligonucleotide sequences of the adaptors and primers. | | |
| --- | --- | --- |
| **Sequence (5**′**–3**′**)** | | |
| Adaptors | | |
| Ada1a | ACACTCTTTCCCTACACGACGCTGTTCCGATCTNNN |  |
| Ada1b | AGATCGGAACAGC |  |
| Ada2a | GTGACTGGAGTTCAGACGTGTGCTCTTCACGANNN |  |
| Ada2b | TCGTGAAGAGCAC |  |
| Ada3a | ACACTCTTTCCCTACACGACGCTCTTCATCGNNN |  |
| Ada3b | CGATGAAGAGCGT |  |
| Ada4a | GTGACTGGAGTTCAGACGTGTGCTCTTCAGCANNN |  |
| Ada4b | TGCTGAAGAGCAC |  |
| Ada5a | ACACTCTTTCCCTACACGACGCTCTTCATGCNNN |  |
| Ada5b | GCATGAAGAGCGT |  |
| Ada6a | GTGACTGGAGTTCAGACGTGTGCTCTTCAGACNNN |  |
| Ada6b | GTCTGAAGAGCAC |  |
| Ada7a | ACACTCTTTCCCTACACGACGCTCTTCAGTCNNN |  |
| Ada7b | GACTGAAGAGCGT |  |
| Ada8a | GTGACTGGAGTTCAGACGTGTGCTCTTCACAGNNN |  |
| Ada8b | CTGTGAAGAGCAC |  |
| Ada9a | ACACTCTTTCCCTACACGACGCTCTTCACTGNNN |  |
| Ada9b | CAGTGAAGAGCGT |  |
| Ada10a | GTGACTGGAGTTCAGACGTGTGCTGTTCCGATCTNNN |  |
| Ada10b | AGATCGGAACAGC |  |
| Primers | | |
| Prim1 | ACACTCTTTCCCTACACGACGCT |  |
| Prim2 | GTGACTGGAGTTCAGACGTGTGCT |  |
| BioPrim1 | (biotin)-ACACTCTTTCCCTACACGACGCT |  |
| BioPrim2 | (biotin)-GTGACTGGAGTTCAGACGTGTGCT |  |
| I5 index primer | AATGATACGGCGACCACCGAGATCTACACNNNNNNACACTCTTTCCCTACACGACGCTCTTCCGATCT |  |
| I7 index primer | CAAGCAGAAGACGGCATACGAGATNNNNNNGTGACTGGAGTTCAGACGTGTGCTCTTCCGATCT |  |

**Table 4 Volume for PCR**

| Component | Volume (for single tag; µl) |
| --- | --- |
| Digestion product | 18 |
| 5×HF buffer | 10 |
| 10 Mm dNTP | 1.2 |
| Primer A (10 µM ) | 0.8 |
| Primer B (10 µM ) | 0.8 |
| Phusion high-fidelity DNA polymerase (2 U/µl) | 0.4 |
| Pure water | 18.8 |
| Total | 50 |

**Table 5 The primer combinations for the five tag positions**

| Tag position | Primer A | Primer B |
| --- | --- | --- |
| 1 | Prim 1 | BioPrim2 |
| 2 | BioPrim1 | BioPrim2 |
| 3 | BioPrim1 | BioPrim2 |
| 4 | BioPrim1 | BioPrim2 |
| 5 | BioPrim1 | Prim 2 |
