# Supplementary material for: Screening and characterization of sex-specific sequences through 2b-RAD sequencing in American shad (Alosa sapidissima)
Source: PLoS One. 2023 Mar 2;18(3):e0282165. doi: 10.1371/journal.pone.0282165 (PMC9980781; doi:10.1371/journal.pone.0282165)
Supplement: S2 Text — (DOCX) [file pone.0282165.s003.docx]

The total PCR volume was 20 μl, with 1 μl of template DNA (0.1 µg), 10 μl of 2 × Taq Plus Master Mix II (Dye Plus) (Vazyme, American), 1 μl of Primer 1 (10 µM), 1 μl of Primer 2 (10 µM), and 7 μL of ddH_2_O (primers were shown in Table 1). A 96-well thermal cycler (T100, Bio-Rad) was used for PCR using the following program: denaturation at 95 °C for 3 min, followed by 35 cycles of denaturation at 95 °C for 15 s, annealing at 66/70 °C for 30 s, and extension at 72 °C for 30 s, and a final extension at 72 °C for 5 min. PCR products were separated through electrophoresis in 1.5% agarose gels using the DM2000 DNA marker (Takara, Japan) as the standard DNA ladder.

**Table 1** **Volume for PCR**

| Component | Volume (for single tag; µl) |
| --- | --- |
| Digestion product | 10 |
| 10× T4 ligase buffer | 1 |
| 10 m M ATP | 1 |
| Adaptor A (5µM) | 0.8 |
| Adaptor B (5µM) | 0.8 |
| T4 DNA ligase (400 U/µl) | 0.5 |
| Pure water | 5.9 |
| Total | 20 |
