# Supplementary material for: Screening and characterization of sex-specific sequences through 2b-RAD sequencing in American shad (Alosa sapidissima)
Source: PLoS One. 2023 Mar 2;18(3):e0282165. doi: 10.1371/journal.pone.0282165 (PMC9980781; doi:10.1371/journal.pone.0282165)

1. The loading order of raw images is marked in the text. Which figure panel was generated from that original image is also marked in raw images.
2. Experimental samples of all the raw images are DNA of American Shad.
3. Method used to capture the image: We used the gel imaging system (QuickGel 6200, Monad, China) to take pictures.

The method was as follows：

Operation Guide

1. Take out the instrument, place it on a horizontal table, connect the power cable and data transmission cable, and make sure that the instrument is on the "O" side.
2. Press the power switch to the "I" side and start it to use.
3. Open the fuselage compartment door and place the gel on the specimen stage (Before placing the albumen glue, pull down the white light board on the inner side of the fuselage.) Click Preview to check the placement of the gel. If no problem exists, turn on the transmissive ultraviolet/transmissive white light source for automatic exposure. After the exposure is complete, click Collect to obtain pictures. Click Save to save the obtained pictures to the selected location. If the automatic exposure picture cannot meet the requirement, click "〈"，"〉"fine adjustment or "《", "》" to preview the transmission white light, capture the transmission ultraviolet day and save the coarse exposure time, or click the digital pop-up keyboard to directly input the target value, or manually adjust the contrast. Optimized the image exposure effect.
4. After adjusting the image, click Save to save the image.
5. If you want to shoot the next gel or shoot again after the shooting is complete, click the "Stop" button. When the "Preview" button turns red, select the light source to continue the exposure.
6. After using, power off the monitor, and then power off the power button on the rear side of the phone.

Raw images

Original image 1

Fig2.a was generated from that original image 1.


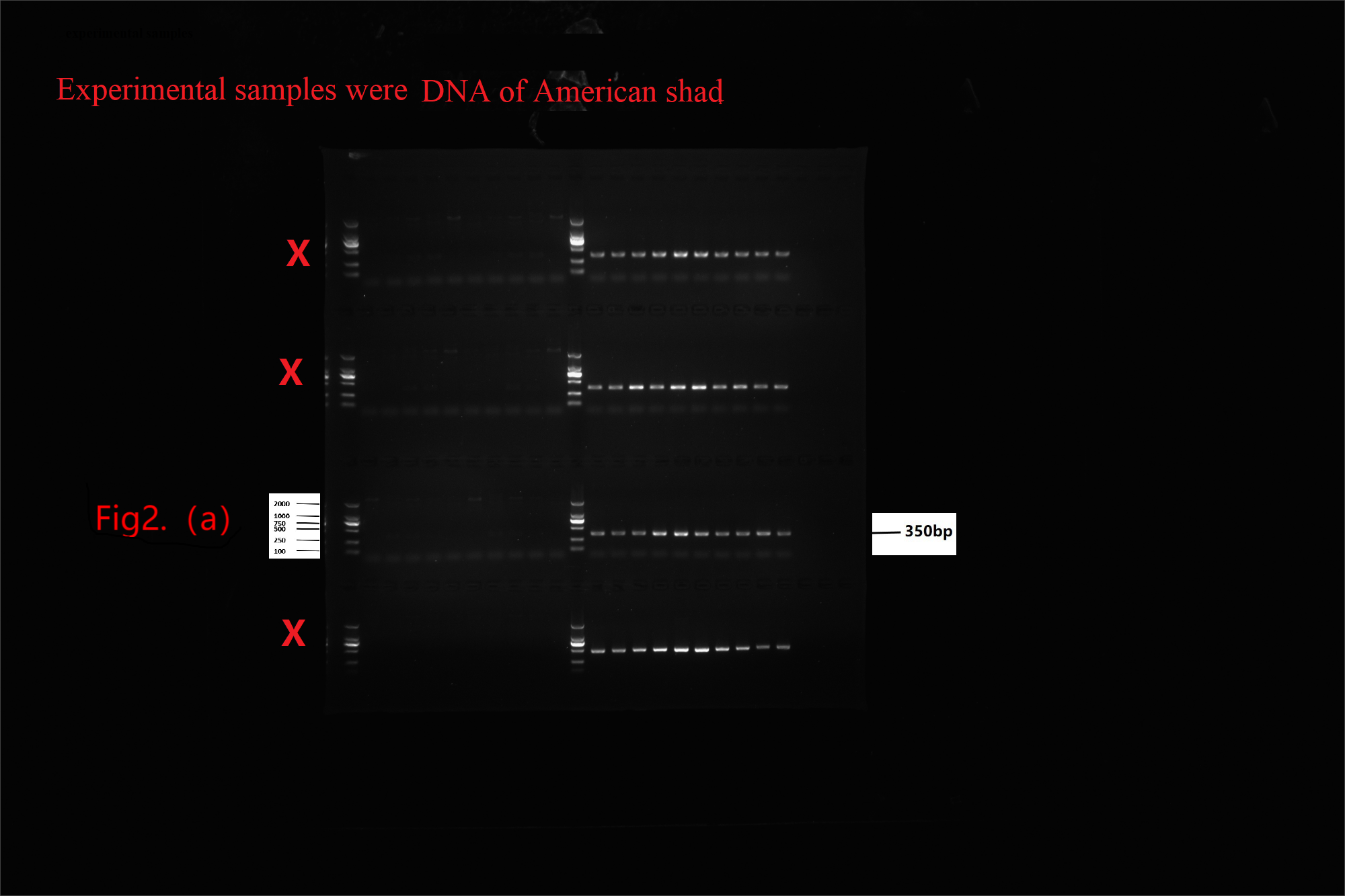


Original image 2

Fig2.b was generated from that original image 2.


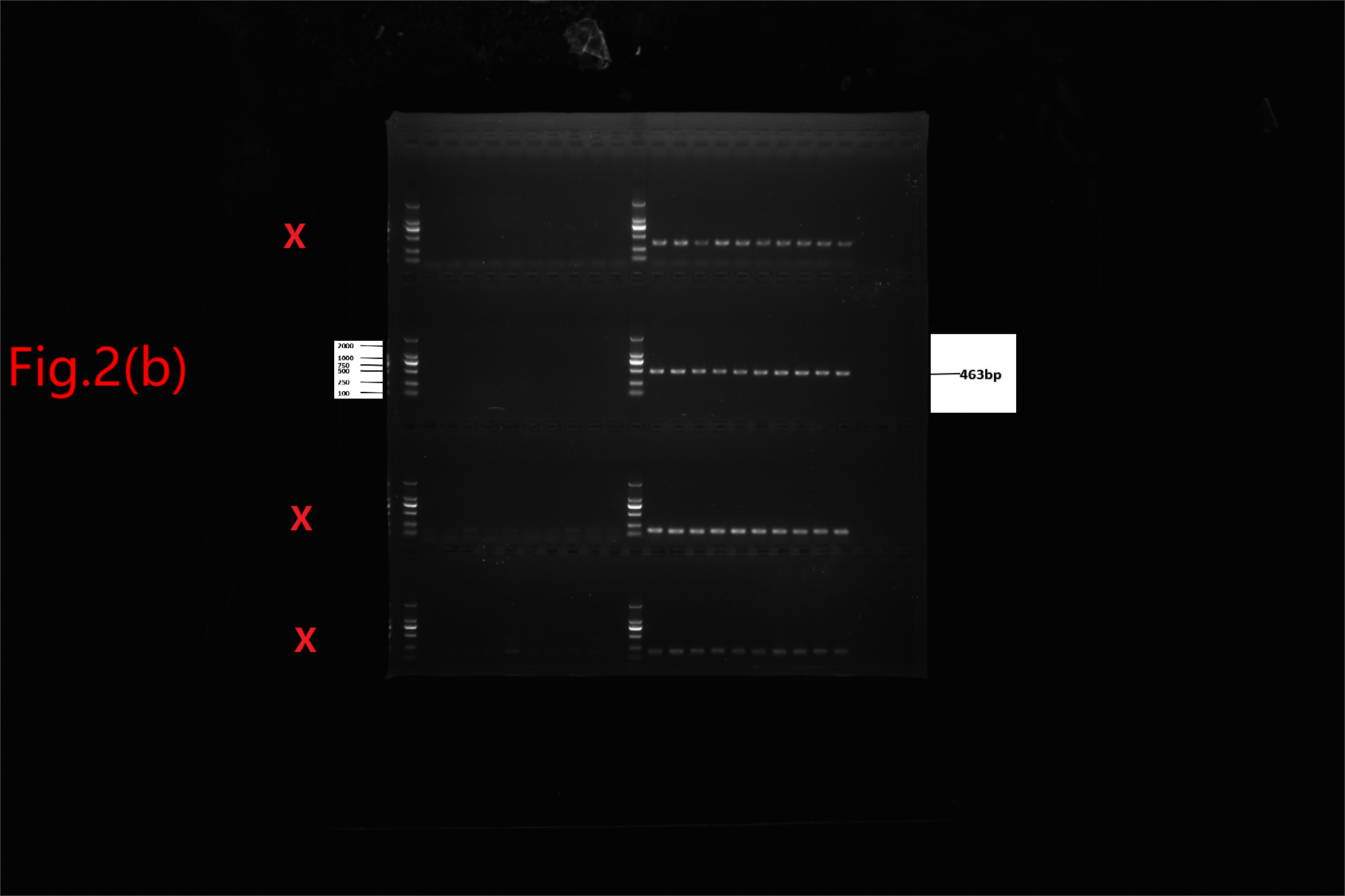


Original image 3

Fig2.C was generated from that original image 3.


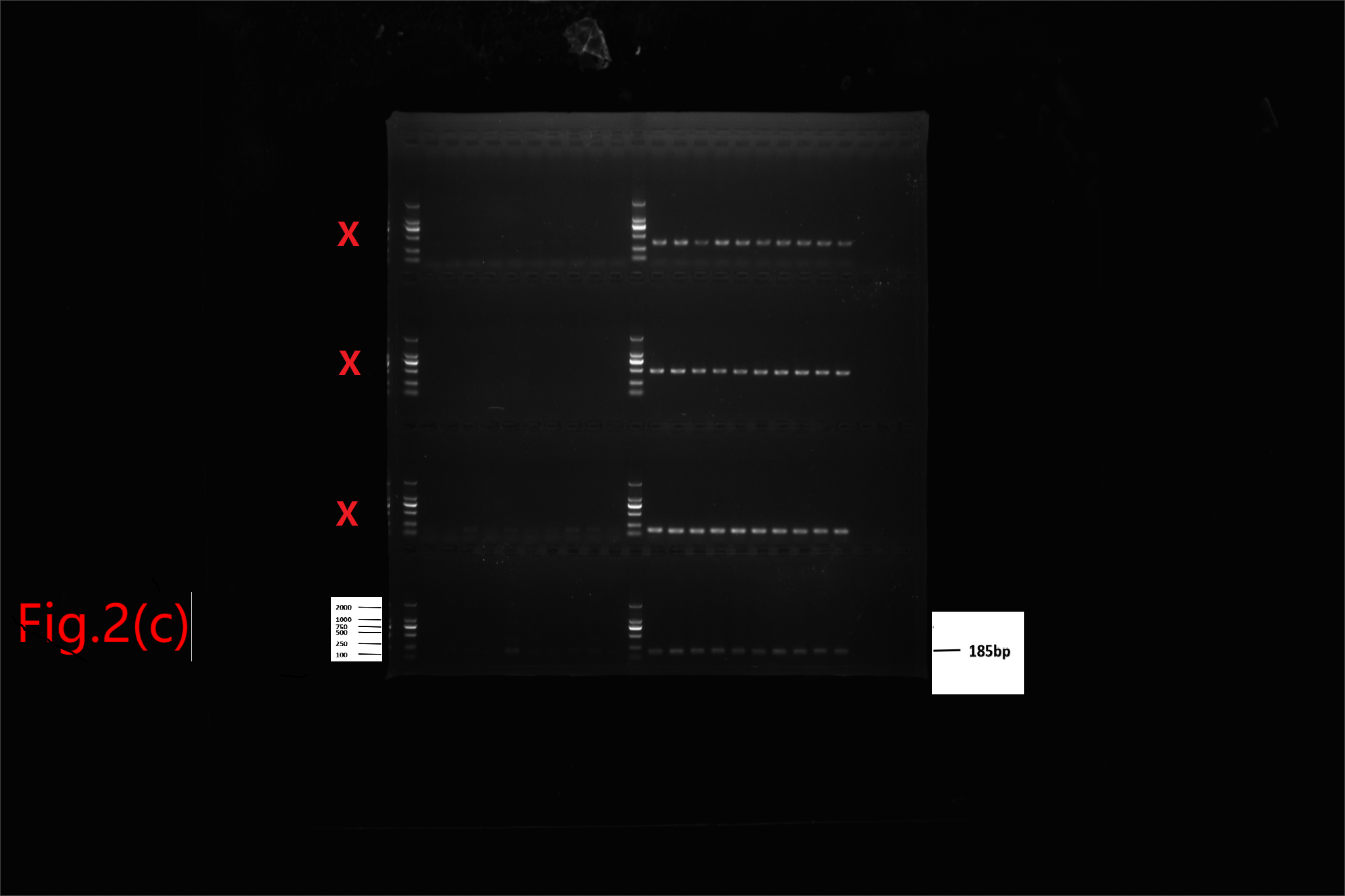


Original image 4

Fig2.D was generated from that original image 4.


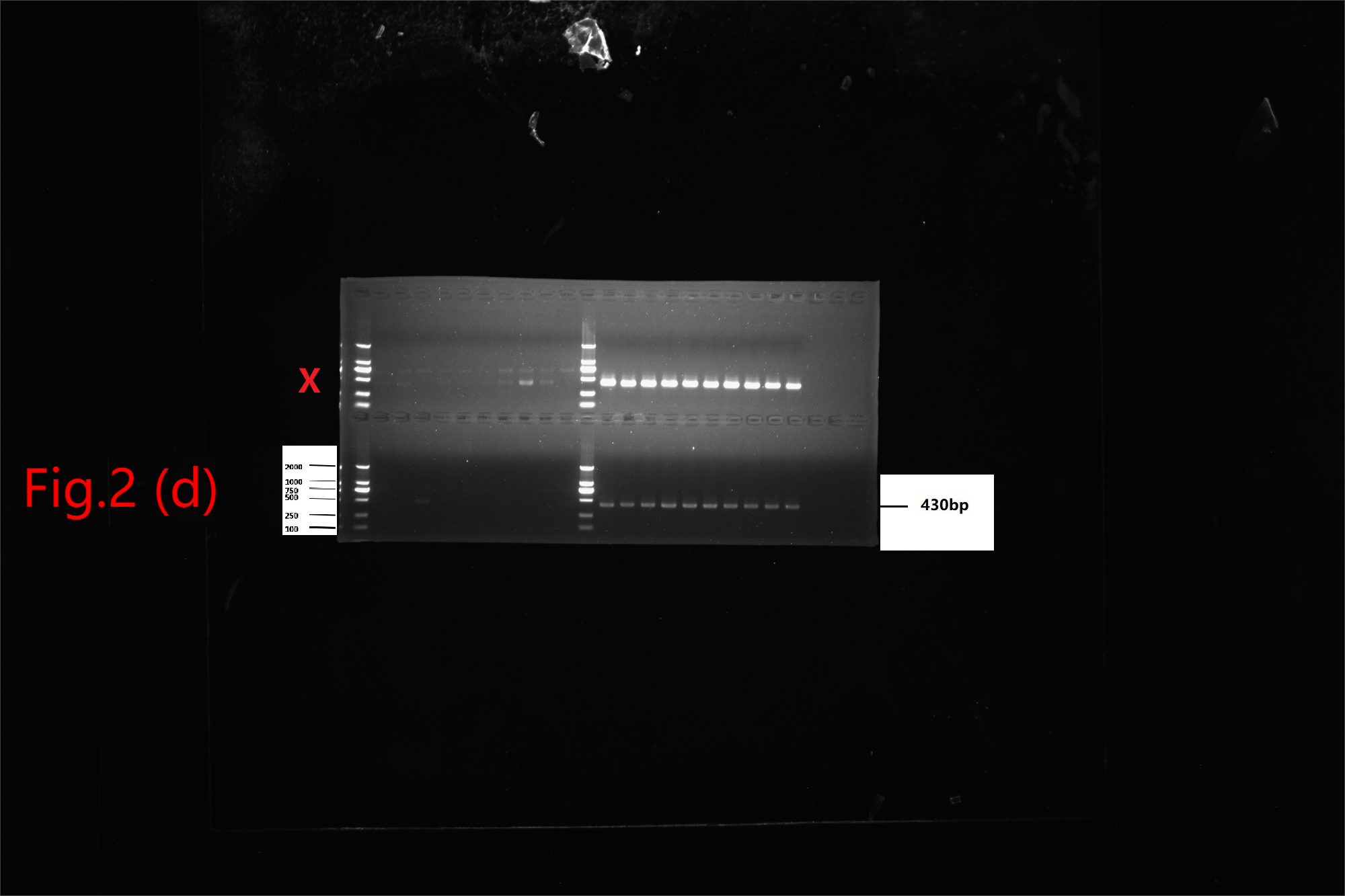


Original image 5

Fig2.E was generated from that original image 5.

**
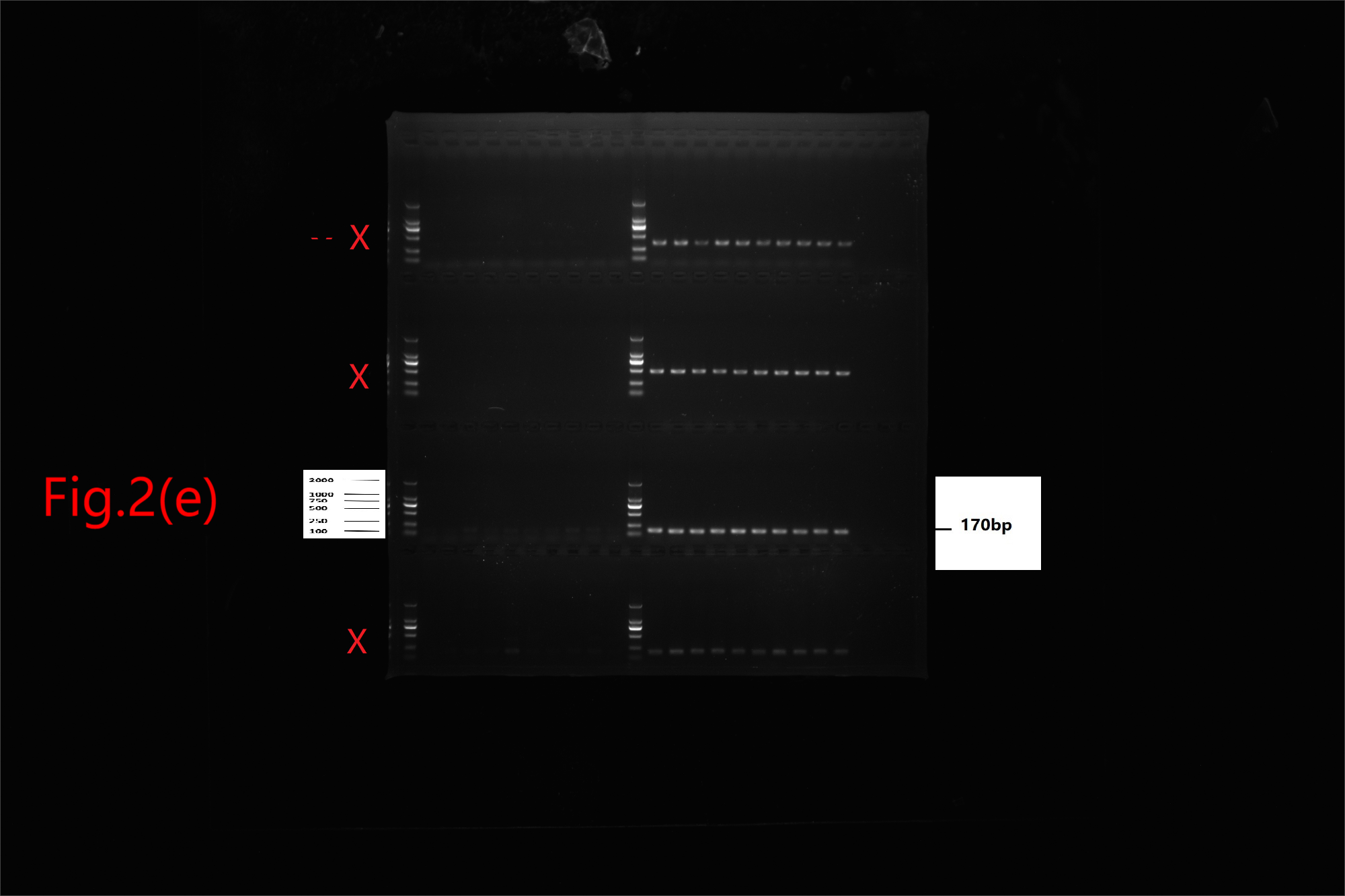
**

Original image 6

Fig3.A was generated from that original image 6.


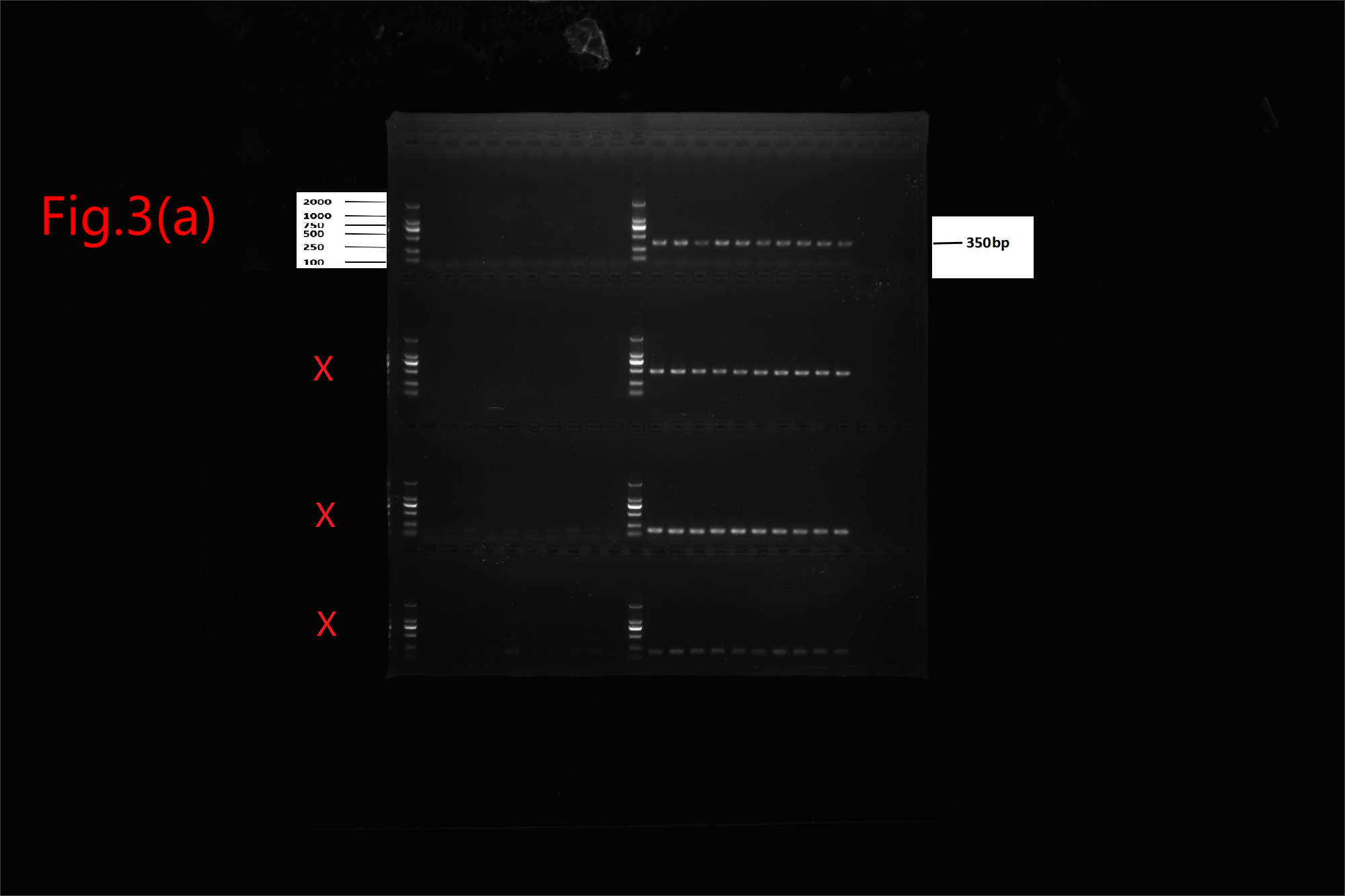


Original image 7

Fig3.B was generated from that original image 7.


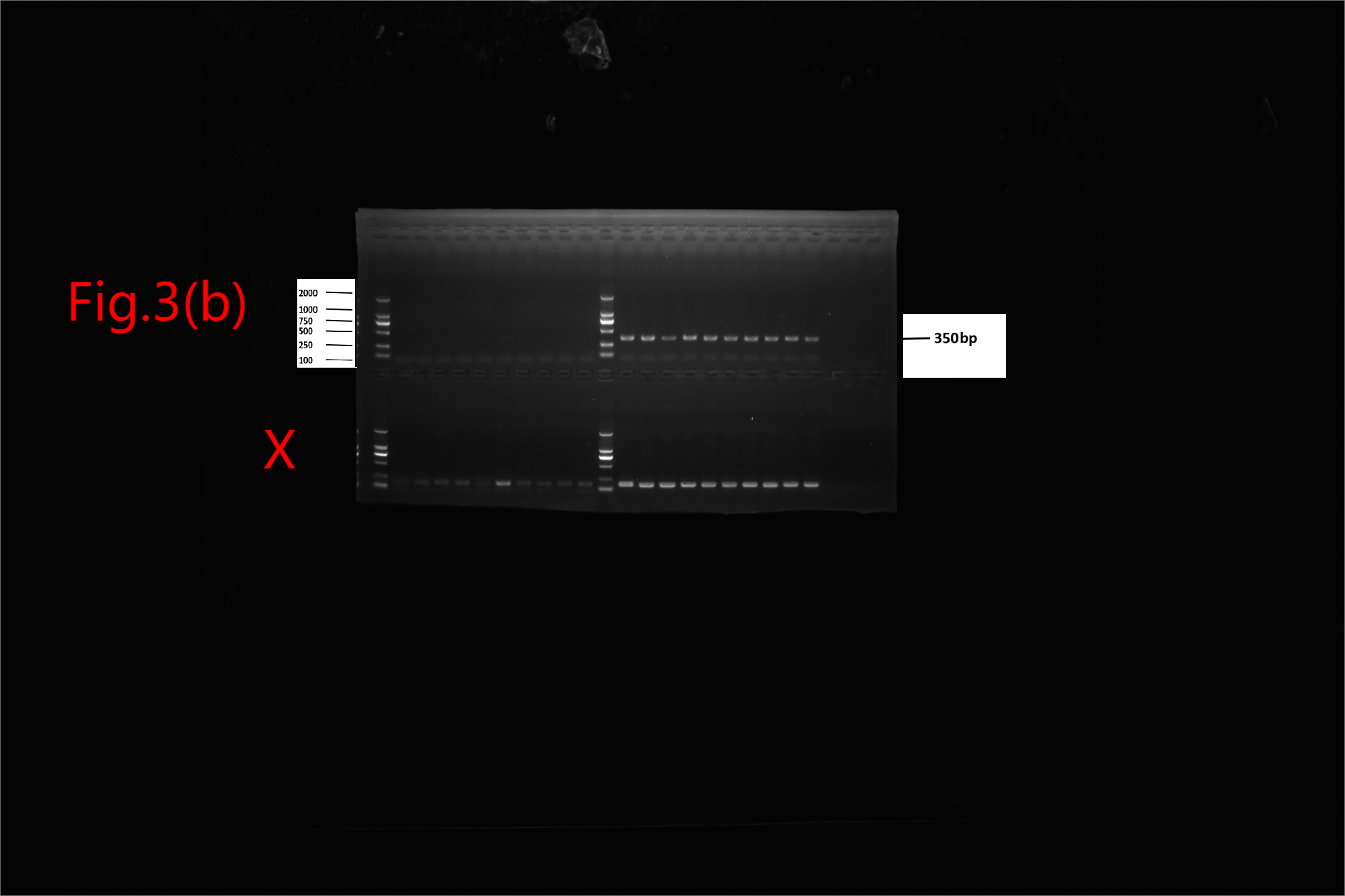


Original image 8

Fig4.A was generated from that original image 8.


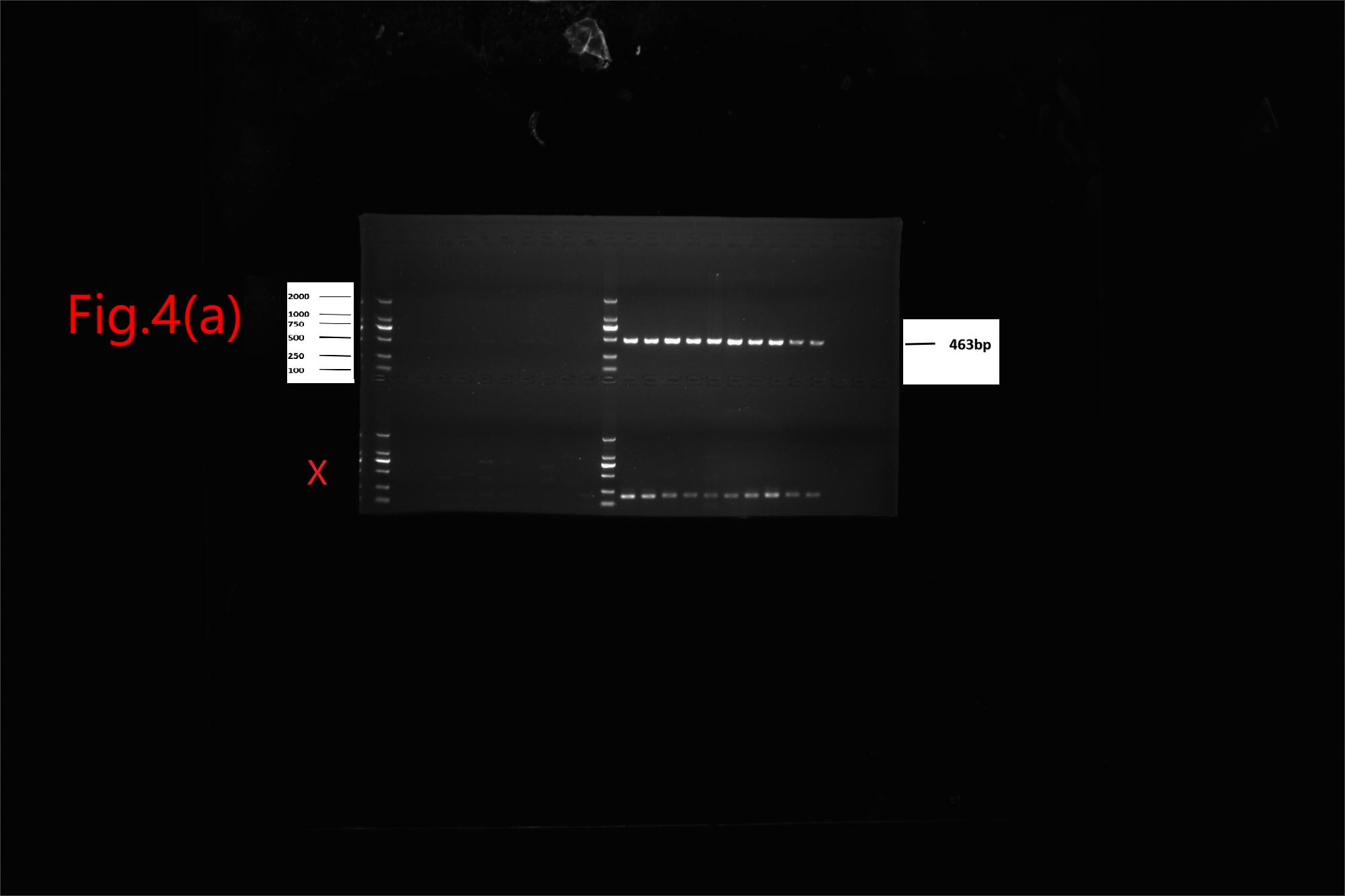


Original image 9

Fig4.B was generated from that original image 9.


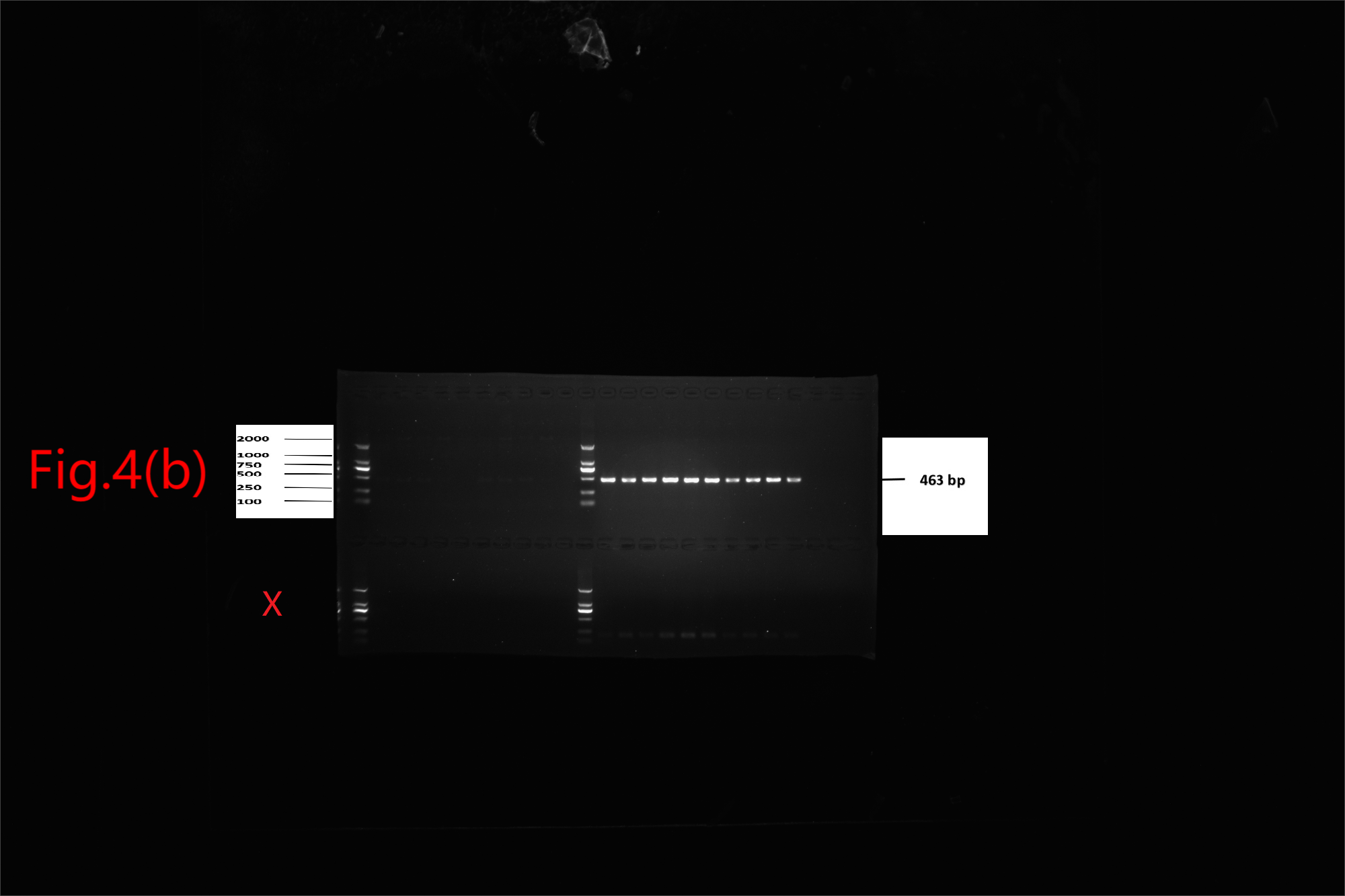


Original image 10

Fig5.A was generated from that original image 10.


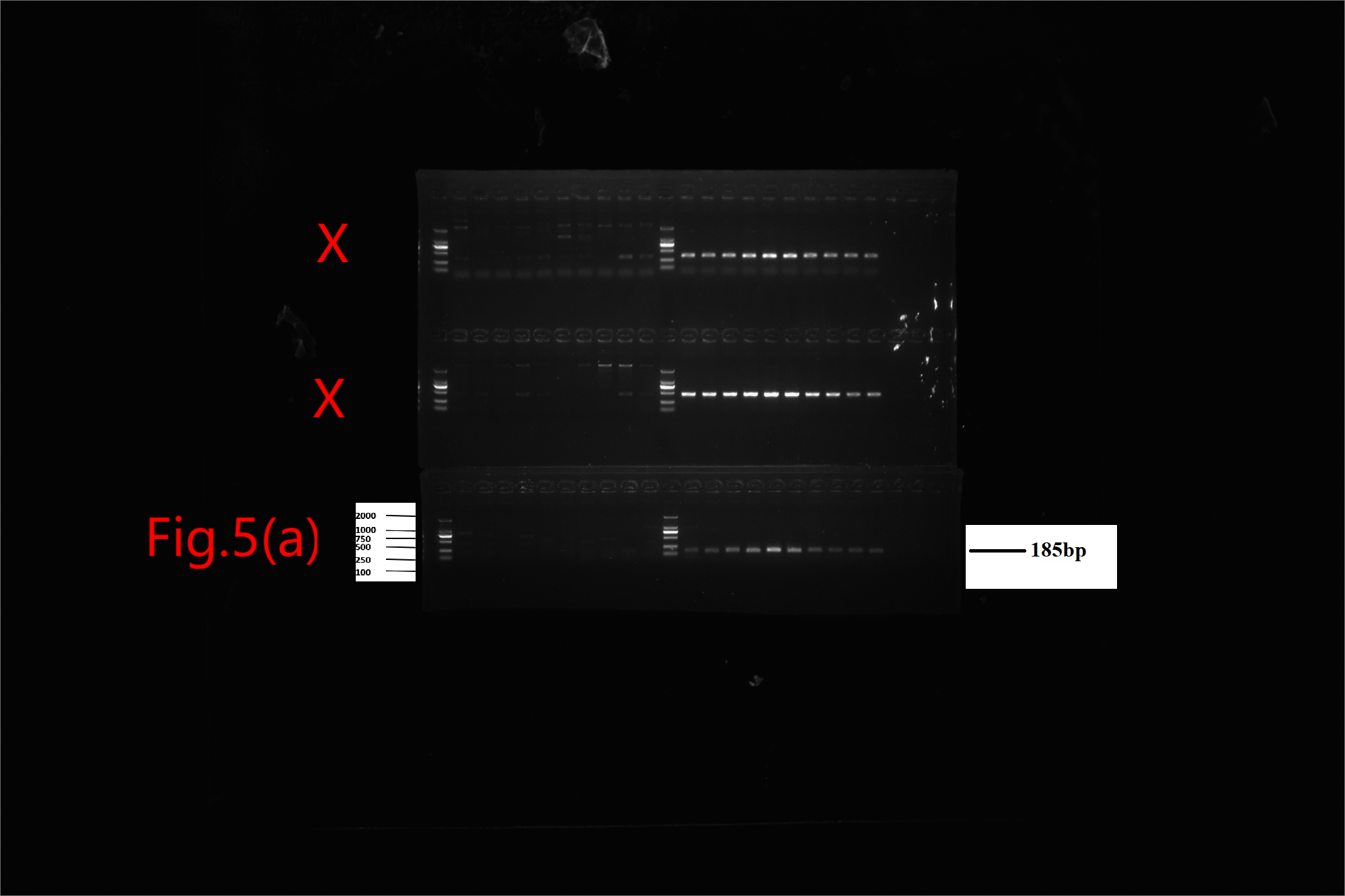


Original image 11

Fig5.B was generated from that original image 11.


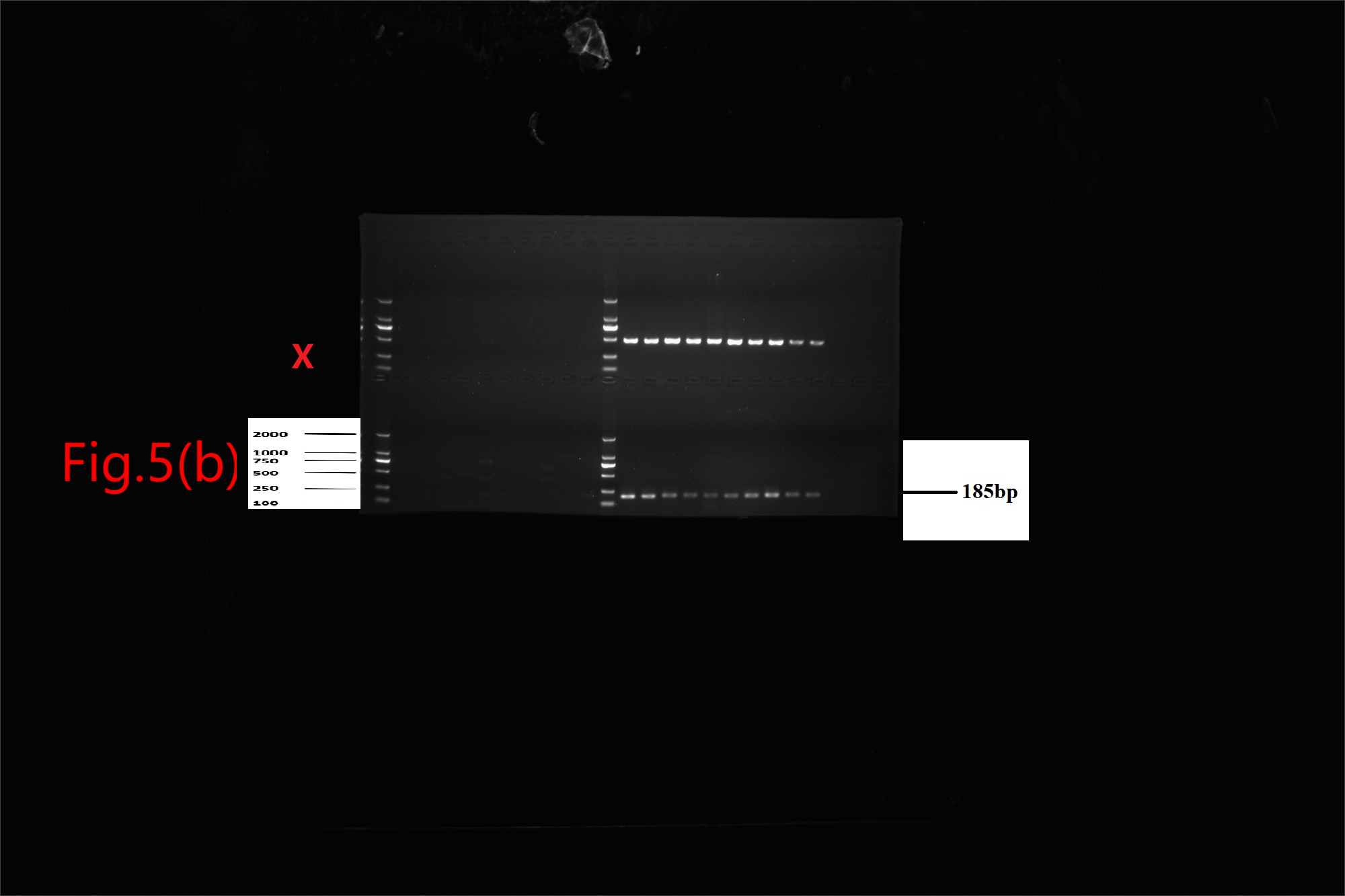


Original image 12

Fig6.A was generated from that original image 12.


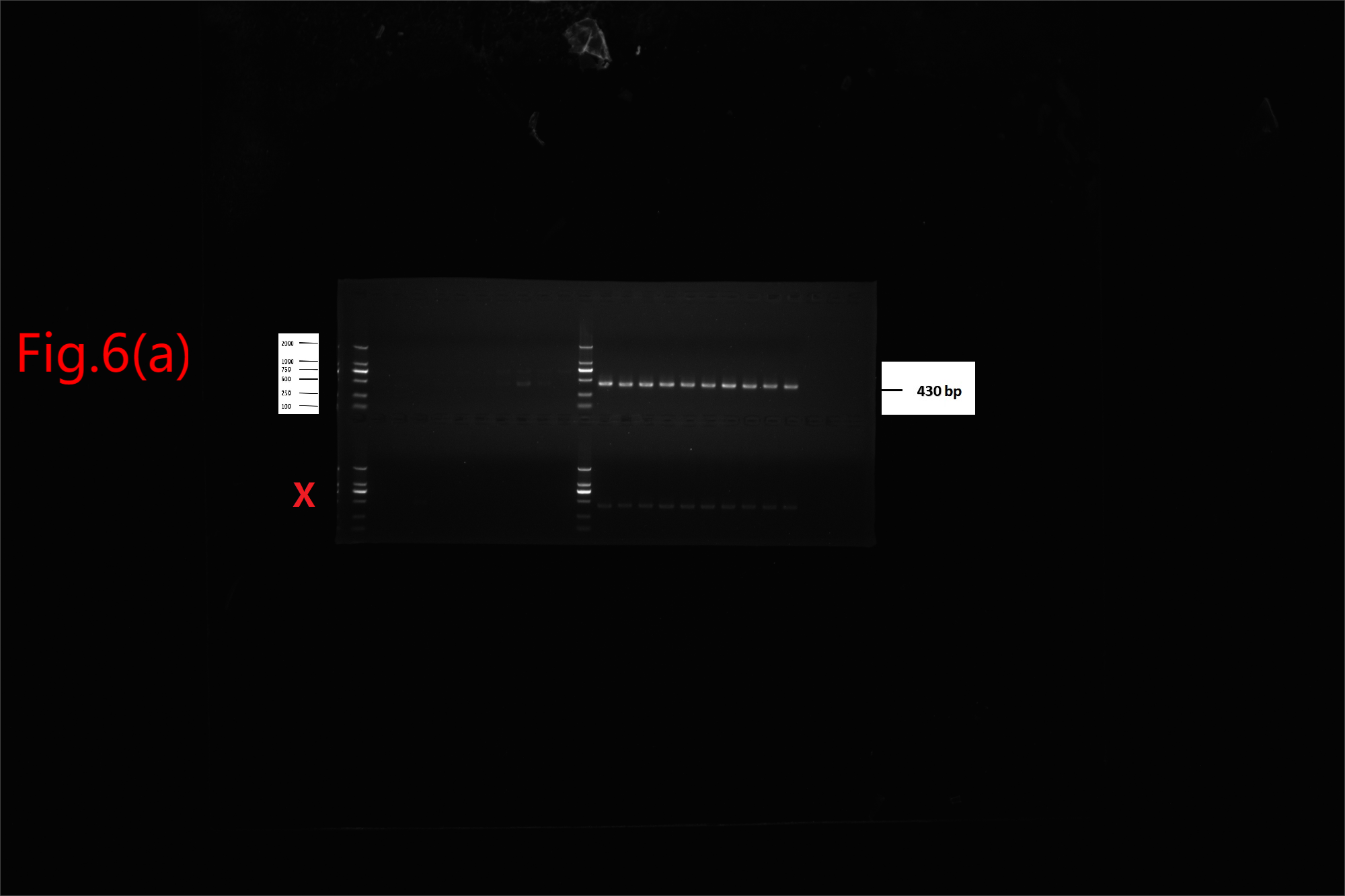


Original image 13

Fig6.B was generated from that original image 13.


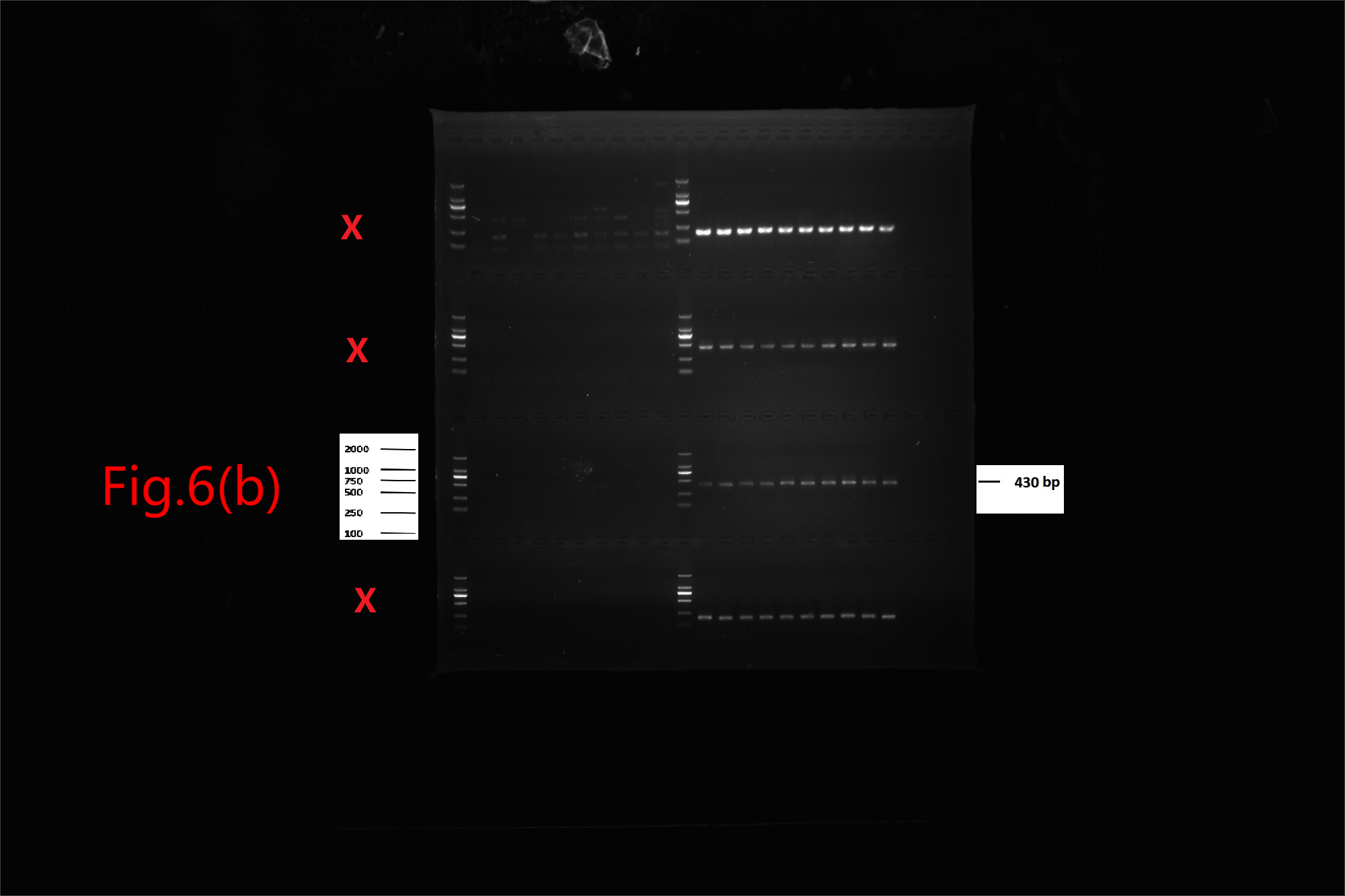


Original image 14

Fig7.A was generated from that original image 14.


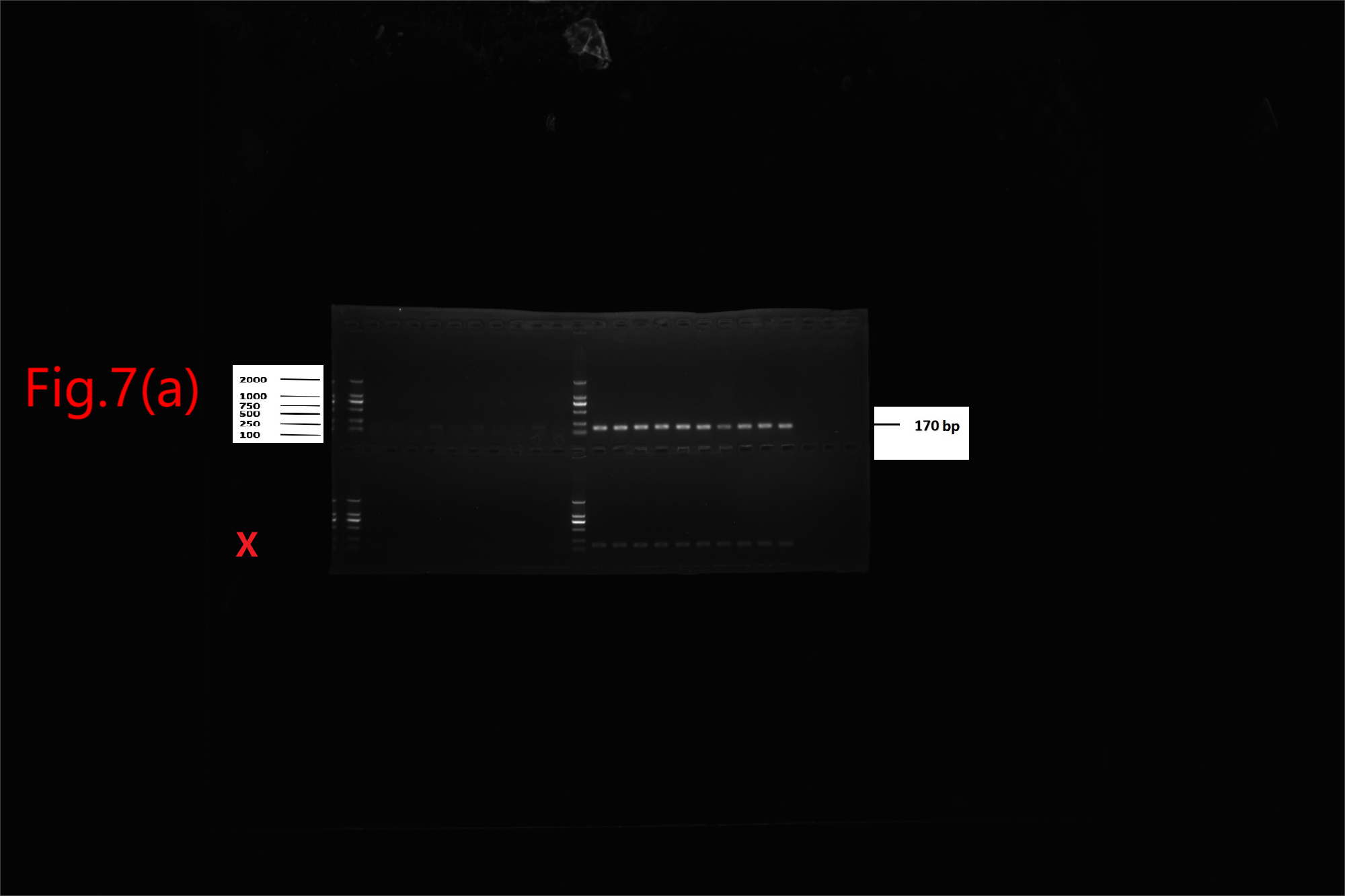


Original image 15

Fig7.B was generated from that original image 15.


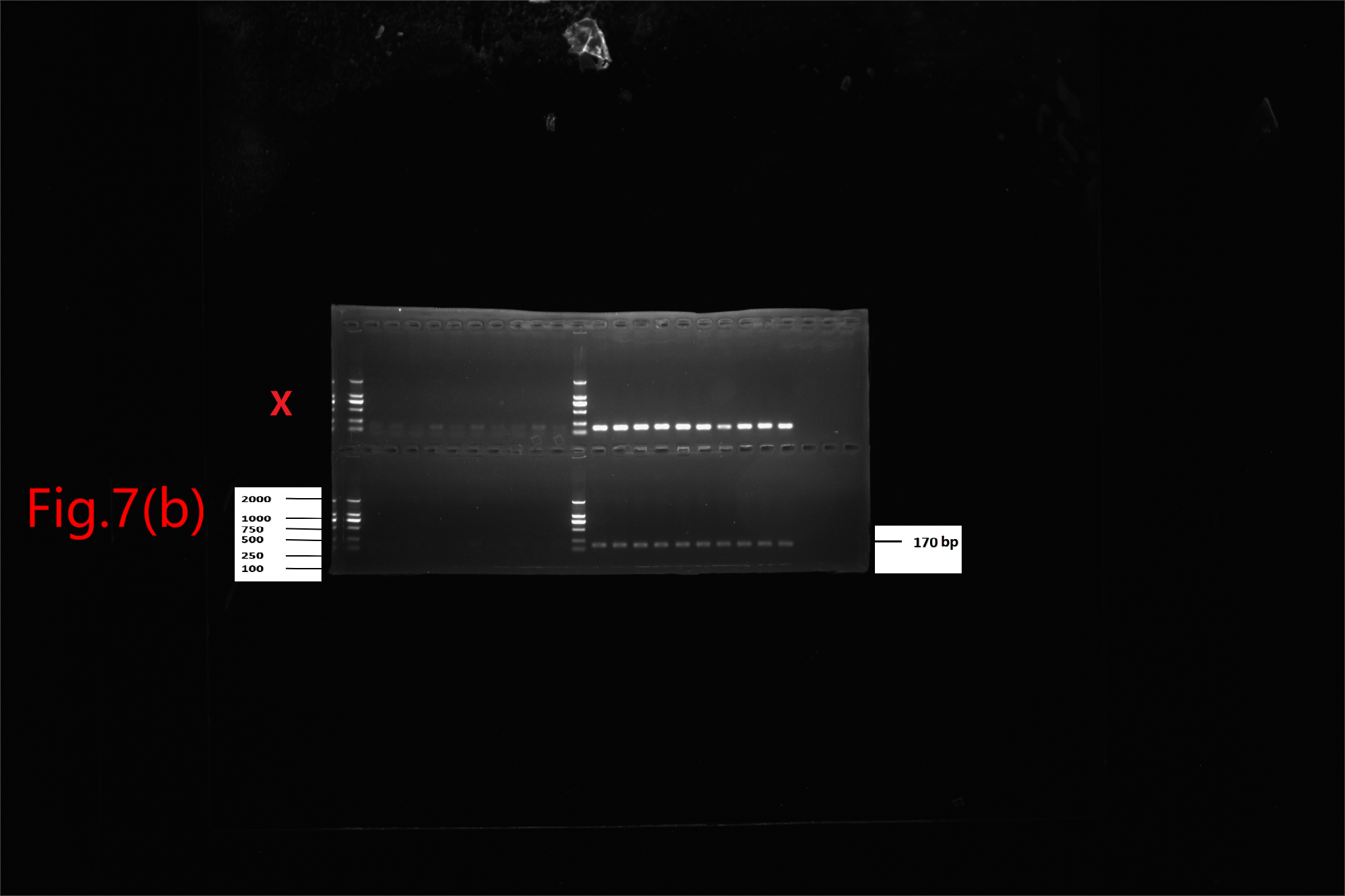

Supplement: S1 Raw images — (DOCX) [file pone.0282165.s004.docx]
